# A Review of Potential Public Health Impacts Associated With the Global Dairy Sector

**DOI:** 10.1029/2019GH000213

**Published:** 2020-02-13

**Authors:** Leah Grout, Michael G. Baker, Nigel French, Simon Hales

**Affiliations:** ^1^ Department of Public Health University of Otago Wellington New Zealand; ^2^ School of Veterinary Science, Hopkirk Research Institute Massey University Palmerston North New Zealand

## Abstract

Strong demand for dairy products has led to a global increase in dairy production. In many parts of the world, dairy systems are undergoing rapid intensification. While increased production may contribute to food security, higher dairy stocking rates in some regions have resulted in increased pressure on natural resources with the potential to affect public health and wellbeing. The aim of this review was to identify and describe the potential health harms and benefits associated with dairy production and consumption. Electronic databases Medline, Embase, Scopus, Web of Science, PubMed, and Google Scholar were searched for published literature that investigated human health impacts of dairy production and consumption. Occupational hazards, environmental health impacts, ecosystem health impacts, foodborne hazards, and diet‐related chronic diseases were identified as potential public health hazards. Some impacts, notably climate change, extend beyond directly exposed populations. Dairy production and consumption are also associated with important health benefits through the provision of nutrients and economic opportunities. As the global dairy sector increases production, exposure to a range of hazards must be weighed with these benefits. The review of impacts presented here can provide an input into decision making about optimal levels of dairy production and consumption, local land use, and identification and management of specific hazards from this sector. Future research should consider multiple exposure routes, socioeconomic implications, and environmental factors, particularly in regions heavily dependent on dairy farming.

## Key Points

- Strong demand for dairy products has led to global growth in production through farmland expansion and more intensive farming practices
- Impacts include exposure to occupational, environmental, and foodborne hazards, and diet‐related harms
- The range of harms must be weighed with benefits to food security, nutrition, livelihoods, and economic opportunities

## 1 Introduction

Dairy production and consumption can have both positive and negative human health effects (Hawkes & Ruel, 2006). Dairy products are major sources of high‐quality protein and bioavailable nutrients (e.g., calcium) (IFPRI, Todd, et al., 2006). Dairy production can also contribute to local‐, regional‐, and national‐level economies and provide opportunities for employment and income generation (IFPRI, Hawkes, et al., 2006), which are critical determinants of health (Marmot et al., 2008). However, a number of potential health harms associated with dairy production and consumption have also been identified, including diet‐related chronic diseases, environmental change, foodborne hazards, occupational hazards, and zoonotic diseases (Horrigan et al., 2002; IFPRI, Hawkes, et al., 2006; Kimman et al., 2013; WHO, 1992).

Globally, there is strong demand for milk and dairy products (FAO & IFCN, 2010; IDF, 2016). This is largely due to global population growth (IDF, 2016), although increases in per capita dairy intake have also driven global demand (FAO and IFCN, 2010). As demand for food increases, agricultural sectors have sought to increase production to meet that demand, and the dairy sector is no exception. In 2014, more than 655 million tons of milk were produced by the global dairy sector (FAO, 2017), and global production is projected to increase by 23% from 2014 to 2025 (OECD & FAO, 2016).

With strong demand for dairy products leading to global and regional increases in dairy production (FAO & IFCN, 2010; IDF, 2016), and with dairy systems in many parts of the world undergoing rapid intensification (OECD & FAO, 2016), concerns have been raised about the implications of intensification for public health and the environment. While increased production may contribute to food security and livelihoods, it may also be associated with a range of health hazards.

This broad review was undertaken in an effort to provide a comprehensive overview of the linkages between the dairy sector and public health. Specifically, the review aims to identify the potential public health impacts associated with dairy production and consumption globally. Both public health hazards and benefits are outlined. Reviewing the health impacts associated with dairy production and consumption will enhance understanding of the potential consequences associated with the intensification of the dairy sector. To the authors' knowledge, no other comprehensive reviews of the potential health impacts associated with dairy production and consumption have been published.

The content of this review could be used to support improved decision making for the future development of the dairy sector, from a public health perspective. Such decisions include

1. Strategic planning for optimal levels of dairy production and consumption at national and global levels and comparison of dairy production and consumption scenarios with plausible alternatives;
2. Strategic planning for the optimal extent of dairy farm development and land use at the national and regional levels and decisions about specific dairy conversion proposals compared with alternative land use options;
3. Prioritization of potential health hazards associated with the dairy sector that require specific risk communication and management actions;
4. Resource allocation for the management of specific hazards associated with dairy production and consumption; and
5. Identification of knowledge gaps that require further research to improve understanding and management of the public health impacts associated with dairy production and consumption.

There are several methods that can be used to support these decision‐making processes by providing systematic assessments of the public health impacts of dairy production and consumption at a wide range of spatial and temporal scales and with varying levels of detail. These methodologies include health impact assessment, health risk assessment, and environmental burden of disease analysis (Grout et al., 2018). All of these processes have a scoping phase to identify relevant hazards to be included. This current review could assist this process by providing a comprehensive list of potential health effects to consider.

## 2 Materials and Methods

### 2.1 Literature Search

The electronic databases Medline, Embase, Scopus, Web of Science, PubMed, and Google Scholar were searched for peer‐reviewed, published literature that investigated the association of dairy production and consumption with potential public health impacts. Search strategies are provided in the supporting information (Texts S1–S5). Articles in languages other than English were excluded. Publication dates were not restricted.

After searching each database, individual article titles and abstracts were assessed to determine their relevance to the topic of this review. Articles focusing on species other than cattle (e.g., goats, sheep, buffaloes, camels, and yaks) or examining animal health impacts rather than human health impacts were excluded. Articles solely relating to beef cattle production and meat consumption were also excluded, despite overlap between the beef and dairy sectors. Furthermore, this review did not include consideration of the impacts associated with dairy sector by‐products, such as the trade in bobby calves; nor did this review assess potential impacts associated with the sale of specialized dairy products, such as those derived from colostrum.

Following the initial full‐text review, additional reports and articles were added to supplement previously obtained information. For example, the search strategies initially utilized for this review were not designed to specifically examine indirect environmental or ecosystem health impacts associated with dairy farming, and only a relatively small sample of relevant articles were originally identified. Therefore, additional articles and reports were added to the review in order to provide a more comprehensive overview of the potential environmental and ecosystem health risks. Where the literature search did not yield sufficient details about effects specific to the dairy sector, additional references about livestock production and consumption in general were used.

### 2.2 Structure of Review

**Figure 1:**
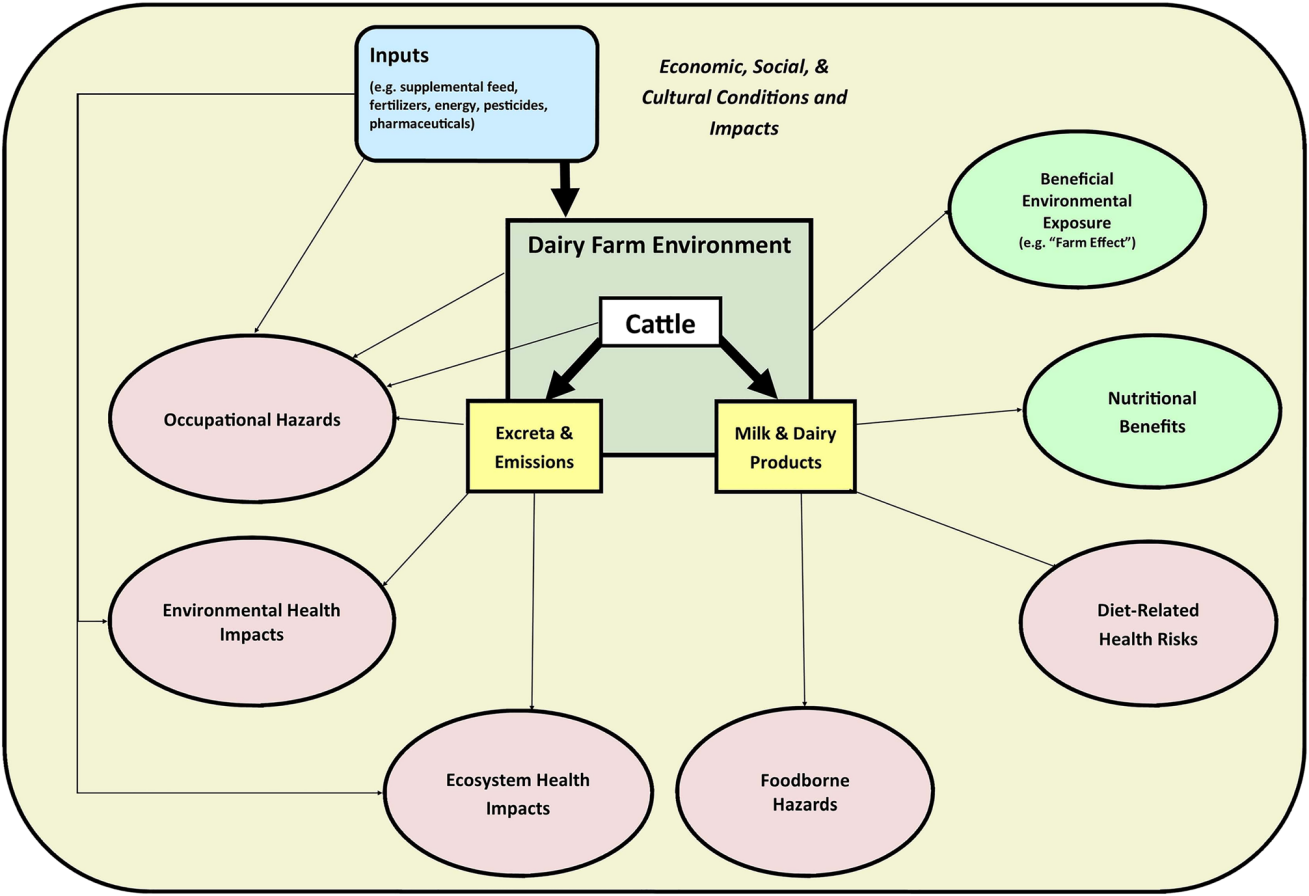
Potential health hazards and benefits associated with dairy farming.

This review examines the potential health harms and benefits associated with dairy production, as well as effects related to the consumption of dairy products (Figure 1). The impacts have been categorized into six broad domains: (i) occupational impacts, (ii) environmental health impacts, (iii) ecosystem health impacts, (iv) foodborne hazards, (v) diet‐related harms and benefits, and (vi) economic, social, and cultural impacts. These categories are not mutually exclusive as there is substantial overlap between them.

The review attempts to include both direct and indirect impacts. Potential indirect impacts that are environmentally mediated, such as climate change, are discussed within the ecosystem health section. The indirect economic, social, and cultural impacts associated with dairy farming that are not necessarily environmentally mediated are discussed separately.

## 3 Occupational Impacts

There are a number of potential biological, chemical, and physical occupational health hazards associated with dairy production, as well as potential protective effects of certain farm exposures. Occupational impacts identified in the literature included zoonotic diseases, risk related to antimicrobial‐resistant pathogens and genes, respiratory symptoms, altered risk for certain types of cancer, and injuries that largely result in musculoskeletal symptoms. Beyond the risk to farmers, farm workers, and farm families, some of the occupational hazards may also present a broader risk to rural communities.

### 3.1 Biological

#### 3.1.1 Zoonoses

Zoonotic pathogens are an important source of human disease and can be detrimental to public health in terms of morbidity and mortality, as well as socioeconomically (McDaniel et al., 2014). Zoonotic infections can severely undercut livestock productivity and reduce farm revenues (McDaniel et al., 2014; WHO, 2012). Cattle are a known reservoir for a number of different pathogens that can cause human illness (FAO et al., 2006; Toth et al., 2013). It has been estimated that at least 60% of human pathogens and 75% of recent emerging diseases have been zoonotic, although many outbreaks have been of wildlife rather than domestic‐animal origin (FAO et al., 2006; Jones et al., 2008; Taylor et al., 2001). Furthermore, over half of global emerging infectious disease events of zoonotic origin from 1940 to 2005 resulted from changes in land use, agricultural practices, and food production practices (Keesing et al., 2010; Whitmee et al., 2015). In a review of bovine zoonotic diseases, 45 different pathogens were identified (McDaniel et al., 2014). Approximately 69% of the identified bovine zoonoses have a global distribution (McDaniel et al., 2014). Bovine zoonoses can be spread to humans through a variety of different transmission routes, including through cutaneous, inhalation, ingestion, and vector‐borne pathways (McDaniel et al., 2014). A significant portion of bovine zoonotic pathogens also have the ability to transmit from human to human, although most are not highly transmissible and therefore do not typically result in large epidemics (McDaniel et al., 2014).

Dairy farm workers, farm residents, veterinarians, and abattoir workers are at increased risk for zoonotic diseases (McDaniel et al., 2014; Toth et al., 2013). For example, leptospirosis is an important occupational zoonosis and farm workers are at high risk for exposure to *Leptospira* spp. (Cowie & Bell, 2012; McDaniel et al., 2014; Thornley et al., 2002). Other commonly reported bovine zoonotic pathogens include *Salmonella* spp. (Cavirani, 2008; Toth et al., 2013; Whitfield et al., 2017), Shiga toxin‐producing *Escherichia coli* (STEC) (Cavirani, 2008; Toth et al., 2013; Whitfield et al., 2017), *Campylobacter* spp. (Cavirani, 2008; Toth et al., 2013; Whitfield et al., 2017), *Cryptosporidium* spp. (Cavirani, 2008; Toth et al., 2013), *Brucella* spp. (Cavirani, 2008), *Mycobacterium bovis* (Cavirani, 2008), *Listeria* spp. (Cavirani, 2008), *Coxiella burnetii* (Cavirani, 2008), *Trichophyton verrucosum* (Cavirani, 2008), *Yersinia* spp. (Whitfield et al., 2017), and *Giardia* spp. (Whitfield et al., 2017), although epidemiological data, including transmission risk factors and incidence rates, are often unavailable in the existing literature and may vary regionally (McDaniel et al., 2014). Additionally, there are often a number of different reservoir host species (e.g., other domestic animals and wildlife species; Jones et al., 2013; WHO, 2012), and it can be difficult to ascertain human cases resulting specifically from bovine exposure (McDaniel et al., 2014).

It is possible that long‐term or frequent exposure to certain pathogens, which can occur on farms, may confer a degree of immunity (Havelaar et al., 2009). However, the role of innate versus acquired immunity in the epidemiology of specific zoonoses is not well understood (Havelaar et al., 2009; Rothman & Mahon, 2004; Swift & Hunter, 2004) and will not be covered in this review.

#### 3.1.2 Antimicrobial Resistance

Dairy farm workers and those who come into direct contact with cattle or cattle excreta may also be at increased risk for the transmission of antimicrobial‐resistant bacteria or genes (Aitken et al., 2016). The use of antimicrobials in food‐producing animals, including dairy cattle, can improve animal health and increase productivity (Call et al., 2008; Oliver et al., 2011). However, the use of antimicrobials, both in humans and in animals, has driven the emergence of antimicrobial‐resistant pathogens and antimicrobial‐resistant genes (Tripathi & Tripathi, 2017). The extent to which antibiotic use in agriculture has driven the emergence of resistant bacterial strains continues to be a topic of debate (Call et al., 2008; Oliver et al., 2011).

Antimicrobials are used in food animals to treat infections and for prophylaxis (Aitken et al., 2016; Call et al., 2008; Tripathi & Tripathi, 2017). However, food animals may also be given antimicrobials to promote growth and increase feed efficiency (Aitken et al., 2016; Tripathi & Tripathi, 2017), although the use of antimicrobials for growth promotion in food‐producing animals has been banned in the European Union and in other countries (Hillerton et al., 2017). On dairy farms, the majority of antimicrobials are used to treat mastitis (Call et al., 2008). Antimicrobials are also used for the treatment of lameness, respiratory disease, reproductive problems, diarrhea or other digestion issues, and pink eye in cattle (Call et al., 2008; Oliver et al., 2011). Additionally, many dairy farms practice dry cow therapy and use intramammary antibiotics following the last milking of lactation (Oliver et al., 2011). However, dairy cattle production tends to be less reliant on antimicrobials than swine or poultry production (Burgess & French, 2017; Collis et al., 2019; Van Boeckel et al., 2015).

Nonionophore antibiotics have increasingly been found in agroecosystems, including on dairy farms (Campagnolo et al., 2002; Tripathi & Tripathi, 2017; Watanabe et al., 2010; Zhang et al., 2013). However, the presence of antimicrobial‐resistant bacteria on dairy farms seems to depend on a number of factors, and farm management practices may play an important role in the status of a dairy herd. For example, in a cross‐sectional study to evaluate the prevalence of extended‐spectrum beta lactamase (ESBL)‐producing and plasmid‐mediated AmpC‐producing *E. coli* in dairy herds in the Netherlands, researchers found that the use of certain classes of antibiotics, especially third‐ or fourth‐generation cephalosporins, and the treatment of all clinical mastitis cases with antibiotics, were associated with ESBL or AmpC‐positive herd status (Gonggrijp et al., 2016).

Antibiotic‐resistant bacteria in dairy cattle can be transmitted to humans through the environment, food products, or direct contact with animals (Aitken et al., 2016; Call et al., 2008; Oliver et al., 2011; Tripathi & Tripathi, 2017). However, a recent population‐based modeling study in the Netherlands found that most community‐acquired ESBL producing and plasmid‐mediated AmpC‐producing *E. coli* carriage was attributable to human‐to‐human transmission (60.1%, 95% CI 40.0–73.5), while food accounted for 18.9% (95% CI 7.0–38.3) of carriage, companion animals for 7.9% (95% CI 1.4–19.9), farm animals (nonoccupational contact) for 3.6% (95% CI 0.6–9.9), and environmental contact (i.e., swimming in freshwater and wild birds) for 2.6% (95% CI 0.2–8.7) (Mughini‐Gras et al., 2019). While humans may be the main source of community‐acquired ESBL producing and plasmid‐mediated AmpC‐producing *E. coli*, transmission through food products or direct contact with animals may still play a role in the spread of antimicrobial resistance (AMR), and there are still knowledge gaps surrounding the potential transmission routes between dairy cattle, humans, and the environment (Burgess & French, 2017).

The World Health Organization Advisory Group on Integrated Surveillance of Antimicrobial Resistance commissioned a systematic review and meta‐analysis to summarize the effect that interventions to reduce antibiotic use in food‐producing livestock have on the prevalence of antibiotic‐resistant bacteria in both animals and humans (Tang et al., 2017). The results for the meta‐analysis for animal studies showed that for almost all antibiotic classes, bacterial groups, and sample types, the pooled risk of AMR was lower in intervention groups with reduced antibiotic use than in control groups (Tang et al., 2017). Specifically, the meta‐analysis indicated a 10–15% reduction in the pooled prevalence of AMR isolates in the intervention groups compared to the control groups for food‐producing livestock (Tang et al., 2017). Furthermore, there was an absolute risk reduction of 24–32% in the proportion of isolates from livestock that were multidrug resistant in intervention groups compared to control groups (Tang et al., 2017). Meta‐analysis for human studies indicated that the pooled prevalence of antibiotic‐resistant bacteria in humans was 24% lower in intervention groups in which there was a decrease in the use of antibiotics in animals, as compared to control groups (Tang et al., 2017). A stronger association was found for humans that had direct contact with food‐producing livestock, including dairy cattle (Tang et al., 2017). The results consistently suggest that antibiotic‐resistant bacteria can be exchanged between livestock and farm workers (Tang et al., 2017).

#### 3.1.3 Bioaerosols

Bioaerosols containing endotoxins are considered the most important occupational cause of respiratory disease among livestock farmers (Basinas et al., 2013; Kirychuk et al., 2006; Reynolds et al., 2013; Vogelzang et al., 1998). Bioaerosols are an aggregate of air suspended particles that originate from plants, animals, and microbes (Basinas et al., 2013). Bioaerosols, also referred to as organic dusts, can contain inflammatory and allergenic agents (e.g., fungal spores, bacteria, viruses, and pollen) and microbial‐associated molecular patterns, including endotoxins, glucans, and peptidoglycans (Basinas et al., 2013; Dungan, 2010; Sigsgaard et al., 2005). Bioaerosols on farms are often highly contaminated with endotoxins (Basinas et al., 2013). Endotoxin is a component of the outer membrane of Gram‐negative bacteria (Basinas et al., 2013).

Important sources of bioaerosols and endotoxins on farms include animals, animal wastes, feed, and bedding material (Douglas et al., 2018). Exposure to bioaerosols and endotoxins can occur through inhalation, skin contact, or ingestion, but inhalation is the most important exposure route for the respiratory health of farmers (Basinas et al., 2013). Exposure to bioaerosols can result in inflammatory reactions (Basinas et al., 2013; Sigsgaard et al., 2005), and the most commonly reported health effects are respiratory symptoms and lung function impairment, but gastrointestinal distress, fatigue, weakness, and headache have also been reported (Douglas et al., 2018; Douwes et al., 2003). Specifically, bronchial hyperresponsiveness, accelerated lung function decline, chronic obstructive pulmonary disease, wheezing, asthma‐like symptoms, and chronic bronchitis have been associated with endotoxin exposure in farming (Basinas et al., 2013; Eduard et al., 2009; Monsó et al., 2004; Smit et al., 2010; Vogelzang et al., 1998). Acute flu‐like symptoms (i.e., organic dust toxic syndrome, also referred to as pulmonary mycotoxicosis) can also occur with very high levels of exposure (Basinas et al., 2013; Madsen et al., 2012; Smit et al., 2006). Additionally, a number of studies have linked bioaerosol emissions to Farmer's Lung, a potentially fatal disease that is also referred to as hypersensitivity pneumonitis or extrinsic allergic alveolitis (Douglas et al., 2018). A systematic review found that dairy farm workers experience increased rates of lung conditions including chronic obstructive pulmonary disease, asthma, chronic bronchitis, certain forms of cancer, Farmer's Lung, and organic dust toxic syndrome (Reynolds et al., 2013). Studies on lung function in dairy workers have found increased obstructive pulmonary changes (Reynolds et al., 2013).

Dairy farmers are often exposed to bioaerosol and endotoxin concentrations well above recommended thresholds or established exposure limits (Basinas et al., 2013). Generally, for cattle farmers, animal handling, milking, and feeding are the tasks associated with the highest level of exposure to bioaerosols, while feeding is associated with the highest levels of endotoxins, although endotoxin exposure may be higher during milking on large dairy farms with more than 1,000 cows (Basinas et al., 2013). However, farm building characteristics (e.g., type of flooring, ventilation, heating, and maintenance frequency) strongly influences exposure (Basinas et al., 2013). A study of Danish dairy farms found that factors and tasks that increase exposure to bioaerosols included the use of fully automatic milking, moving cattle, handling animal feed and seeds, handling of silos, and distributing bedding (Basinas et al., 2014). The use of rail feed dispensers in farms without fully automatic milking also increased bioaerosol levels (Basinas et al., 2014). Factors and tasks that increased exposure to endotoxins include lower outdoor temperature, the use of slope‐back or back‐flushed slurry systems, milking, distribution of bedding, and handling of feed and seed in barns (Basinas et al., 2014). Overall, automatic milking and manure handling methods were strongly associated with personable inhalable bioaerosols and endotoxin exposure (Basinas et al., 2014). Specifically, workers on farms with automatic milking systems were exposed to bioaerosol levels that were twice as high (*β* = 0.89, *p* = 0.003; where *β* is the regression coefficient for log‐transformed exposure data) as workers on farms with parlor or pipe milking systems (Basinas et al., 2014).

#### 3.1.4 Beneficial Biological Exposures

A number of studies, including several large cohort studies, have shown a decreased prevalence of asthma, atopy, and atopic disorders in children raised on farms (Alfven et al., 2006; Illi et al., 2012; Mazur et al., 2017; Reynolds et al., 2013; Riedler et al., 2001; Wells et al., 2014; Wlasiuk & Vercelli, 2012). The so‐called “farm effect” has been extensively documented, but it is not clear how exposure to a farm environment during childhood might modify the risk for asthma, atopy, and atopic disorders (Mazur et al., 2017). Neither the specific protective factors of farm exposures, nor the underlying immunological mechanisms have been conclusively determined (Douwes et al., 2008). However, evidence suggests that organic dust exposure, and endotoxin specifically, may be responsible for the observed protective effects (Basinas et al., 2013; Portengen et al., 2005; Spierenburg et al., 2017; Von Ehrenstein et al., 2000). However, it has also been suggested that exposure to the microbiological diversity of the farm environment may provide protection through effects on the innate immune system (Ege et al., 2011; Leavy, 2016; Mazur et al., 2017; Stein et al., 2016). A recent study demonstrated that the asthma risk in children decreased as the similarity of the house dust microbiota composition to that of a farm home increased (Kirjavainen et al., 2019). Specifically, the indoor dust microbial composition in farm homes was characterized by high bacterial richness and cattle associated microbes and was distinct from nonfarm homes (Kirjavainen et al., 2019). The results were consistent with the hypothesis that exposure to microbial diversity in the farming environment provides protection against asthma (Kirjavainen et al., 2019).

It has also been hypothesized that endotoxin exposure may explain the low mortality from lung cancer observed in dairy farmers (Laakkonen & Pukkala, 2008; Lange, 2000; Lenters et al., 2010; Mastrangelo et al., 1996). Experimental studies in animals, as well as several trials in humans, have indicated that endotoxin can inhibit tumor initiation and growth (Chicoine et al., 2001; Lenters et al., 2010; Otto et al., 1996; Reisser et al., 2002). However, while some epidemiological studies of agricultural workers have observed lower than expected lung cancer rates, the results have often been attributed to lower smoking rates among farmers (Laakkonen & Pukkala, 2008; Lange, 2000; Lenters et al., 2010). The results have also occasionally been attributed to a form of selection bias commonly referred to as the healthy worker effect (Lange, 2000). The healthy worker effect refers to the fact that when observing a group of workers, the group may be healthier than the population at large due to the self‐selection of individuals capable of performing the required work (Chowdhury et al., 2017).

### 3.2 Chemical Hazards

Agricultural chemicals (e.g., pesticides), gases (e.g., hydrogen sulfide, nitrogen oxides, and ammonia) and volatile organic compounds (VOCs) can pose a health risk to farm workers (Douphrate et al., 2013). Pesticides are commonly used in agricultural production systems and many have been associated with a number of different adverse human health impacts (Bassil et al., 2007; Dich et al., 1997; García, 2003; Mostafalou & Abdollahi, 2013; Nicolopoulou‐Stamati et al., 2016; Sanborn et al., 2007; Weisenburger, 1993). Exposure to pesticides can occur cutaneously, through ingestion, or through inhalation (Nicolopoulou‐Stamati et al., 2016). There is strong evidence that pesticide exposure contributes to both acute and chronic health effects, including dermatological, gastrointestinal, neurological, carcinogenic, respiratory, reproductive and endocrine effects (Bassil et al., 2007; Dich et al., 1997; García, 2003; Mostafalou & Abdollahi, 2013; Nicolopoulou‐Stamati et al., 2016; Sanborn et al., 2007; Weisenburger, 1993). A systematic review found that farmers have elevated rates for leukemia, non‐Hodgkin's lymphoma, multiple myeloma, soft‐tissue sarcoma, and cancers of the lip, stomach, brain, and prostate, which may be linked to pesticide exposure (Blair & Zahm, 1995). However, a large cohort study that followed both male and female Finnish farmers from 1995 to 2005 found that the only form of cancer that dairy farmers had a significantly elevated risk for was cancer of the lip (Laakkonen & Pukkala, 2008). Overall, farmers tended to have a lower cancer incidence than the general population (Blair & Zahm, 1995; Laakkonen & Pukkala, 2008). Additionally, one review indicated that farmers in the United States tend to have lower mortality for all causes combined, heart disease, and cancers of the bladder, liver, colon, esophagus, rectum, and kidney (Blair & Zahm, 1995).

Certain gases and VOCs emitted on dairy farms can cause respiratory symptoms in humans (Beck et al., 2007; Davidson et al., 2018; Donham, 1993; Eduard et al., 2009; Gerasimon et al., 2007; Hair & Strong, 2005; Place & Mitloehner, 2010; Sunesson et al., 2001). On dairy farms, VOCs can be released from animals, animal wastes, fodder, and bedding materials (e.g., sawdust) (Beck et al., 2007; Place & Mitloehner, 2010; Shaw et al., 2007). For example, a number of different chemical compounds, including alcohols, aldehydes, ketones, carboxylic acids, fatty acids, amines, amides, and thiol compounds, are produced when manure is broken down by bacteria in anaerobic processes (Shaw et al., 2007; Sunesson et al., 2001). Additionally, feed storage and handling can contribute significantly to the emission of alcohols, carbonyls, carboxylic acids, and sulfur‐containing species (Yuan et al., 2017). With regard to occupational health, gases of particular concern on dairy farms include hydrogen sulfide, ammonia, and nitrogen oxides. Exposure to high levels of hydrogen sulfide can inhibit cellular aerobic metabolism (Gerasimon et al., 2007; Sunesson et al., 2001) and in severe cases can cause respiratory arrest, anoxic brain injuries, and death (Donham, 1993; Eduard et al., 2009; Gerasimon et al., 2007; Hair & Strong, 2005). Exposure to ammonia can irritate human mucus membranes, eyes, nasal passages, and skin (Sunesson et al., 2001) and exposure has been associated with reduced lung function and chronic obstructive respiratory disease among farmers (Davidson et al., 2018; Donham, 1993; Eduard et al., 2009). Exposure to nitrogen oxides has also been associated with respiratory symptoms and reduced lung function (WHO, 2017). The contribution of gases to air pollution is discussed in further detail in section 4.1.2.

### 3.3 Physical Hazards

Agriculture is one of the most hazardous work sectors globally and dairy farming has been associated with significantly increased risk of injury in a number of different countries (Douphrate et al., 2013). The International Labour Organization estimates that approximately 170,000 of the 355,000 workplace fatalities that occur worldwide each year involve agricultural workers (Douphrate et al., 2013; ILO, 2017). Dairy farm worker injuries and fatalities are often associated with heavy machinery and vehicle operation, livestock handling, and manure management systems (Douphrate et al., 2013). Slips, trips, and falls are also a common mechanism of injury on dairy farms (Douphrate et al., 2013).

A review of occupational injuries and fatalities on dairy farms examined worker safety statistics for Sweden, New Zealand, Australia, the United States, and China and found that machinery was the leading cause of injuries and fatalities on dairy farms (Douphrate et al., 2013). Additionally, livestock handling is inherently hazardous and dairy bulls in particular are very aggressive and dangerous (Douphrate et al., 2013). The milking of cows also presents a risk for injuries, particularly to the wrists, hands, and fingers, due to proximity to cows' hooves (Douphrate et al., 2013). In a survey of large‐herd milking parlor workers, more than 80% of respondents reported being kicked or stepped on by a cow (Douphrate et al., 2016).

### 3.4 Occupational Impacts Summary

Dairy farm workers, as well as others who come into frequent contact with dairy cattle and their environment, including veterinarians and farm families, may be at increased risk for certain diseases and injuries. Dairy farming, and in particular animal handling and milking tasks, can be physically demanding work (Douphrate et al., 2013; Douphrate et al., 2016). Repetitive tasks, awkward postures, and adverse conditions can contribute to the development of musculoskeletal symptoms in farm workers (Douphrate et al., 2016). Furthermore, farm equipment, heavy machinery, agricultural chemicals, and animals can pose a risk to humans if handled inappropriately (Douphrate et al., 2013). Exposure to bioaerosols and endotoxins on farms can result in respiratory symptoms and lung function impairment (Douglas et al., 2018; Douwes et al., 2003). However, evidence indicates that endotoxin exposure also protects against allergic sensitization and allergic asthma (Basinas et al., 2013; Portengen et al., 2005; Spierenburg et al., 2017; Von Ehrenstein et al., 2000). Additionally, endotoxin exposure may protect against lung cancer in dairy farmers (Laakkonen & Pukkala, 2008; Lange, 2000; Lenters et al., 2010; Mastrangelo et al., 1996) and overall, farmers may have a lower cancer incidence than the general population (Laakkonen & Pukkala, 2008). However, contact with cattle, their excretions, or the dairy farm environment are important risk factors for the transmission of a number of different zoonotic pathogens (McDaniel et al., 2014; Toth et al., 2013; Whitfield et al., 2017)and the transmission of antimicrobial‐resistant pathogens or genes (Aitken et al., 2016).

## 4 Environmental Health Impacts

There are a number of different environmental impacts associated with dairy farming, which can have direct or indirect repercussions for human health and wellbeing. Generally, environmental changes and ecosystem damage can lead to direct environmental health impacts (e.g., water shortages and exposure to pollutants), indirect ecosystem‐mediated health impacts (e.g., altered infectious disease risk, decreased food yield, and cultural impoverishment), and indirect socially mediated health impacts (e.g., livelihood loss, population displacement, and conflict). This section describes important direct environmental health impacts associated with dairy farming including air pollution, water pollution, and soil degradation. Ecosystem health impacts are described separately in section 5.

### 4.1 Air Pollution

The global dairy sector emits a number of different air pollutants, including particulate matter, nitrogen oxides, VOCs, ammonia, methane, and carbon dioxide (FAO et al., 2006; Havlikova et al., 2008; OECD, 2004; Place & Mitloehner, 2010; Won et al., 2017) Carbon dioxide, methane, and nitrous oxide also contribute to climate change (FAO et al., 2006; Grossi et al., 2018; Havlikova et al., 2008; OECD, 2004; Place & Mitloehner, 2010; Won et al., 2017). Generally, important sources of air pollutants on dairy farms include emissions from animals, cropping systems, fossil energy use, feed management, and waste (Grossi et al., 2018; Place & Mitloehner, 2010), and air pollutants can contribute both to environmental damage and human health outcomes, either directly or indirectly (Havlikova et al., 2008). Air pollution is now the environmental health hazard with the largest health burden through its contribution to cardiovascular and respiratory disease morbidity and mortality (WHO, 2017). Outdoor air pollution has also been linked to the development of various forms of cancer, including cancers of the lung, urinary tract, and bladder (WHO, 2017).

#### 4.1.1 Particles

Poor ambient air quality in both cities and rural areas was estimated to cause 4.2 million premature deaths worldwide in 2016 (WHO, 2018a). These premature deaths were largely attributable to exposure to particulate matter smaller than 10 μm (PM_10_), which can cause cardiovascular disease, respiratory illnesses, and cancers (Lelieveld et al., 2015; Pope et al., 2002; Townsend et al., 2003; WHO, 2018a). Particulate matter can harm human health even at very low concentrations (WHO, 2018a) and therefore presents an important environmental health risk from dairy farms (Havlikova et al., 2008; Place & Mitloehner, 2010). Particulate matter emissions from dairy farms occur when cattle are moved, during tillage and harvesting activities for feed production, and indirectly through the formation of secondary particulate matter via oxidation of ammonia or other gaseous precursors (Lelieveld et al., 2015; Place & Mitloehner, 2010).

Agricultural emissions play a critical role in the formation of particulate matter smaller than 2.5 μm (PM_2.5_) in certain regions of the world, including the United States, Europe, Russia, and East Asia (Lelieveld et al., 2015). Livestock production has specifically been estimated to account for approximately 8% of total PM_10_ emissions and 4% of total primary PM_2.5_ emissions, but the contribution of livestock production to secondary PM_2.5_ emissions remains unclear (Cambra‐López et al., 2010). While the emission of primary particulate matter from dairy farms is typically much lower than from poultry or pig operations (Cambra‐López et al., 2010; Hristov, 2011), the emission of secondary PM_2.5_ in the presence of ammonia is a major concern (Cambra‐López et al., 2010; Hristov, 2011; Pozzer et al., 2017; Smit & Heederik, 2017). Smaller particles (e.g., PM_2.5_) have a longer atmospheric lifetime than larger particles, which tend to settle more quickly (Cambra‐López et al., 2010; Melse et al., 2009). Therefore, smaller particles can contribute to air pollution on a regional scale, while larger particles tend to contribute to air pollution on a local scale (Cambra‐López et al., 2010; Smit & Heederik, 2017).

Bioaerosols also present an important environmental health risk from dairy farms (Basinas et al., 2013; Douglas et al., 2018; Nygard et al., 2008). Bioaerosols can be produced and transported from animal production facilities (Basinas et al., 2013; Basinas et al., 2014; Douglas et al., 2018; Dungan, 2010) and the application of manure to agricultural fields (Dungan, 2010). Bioaerosols can remain suspended in the air for long periods and can also travel long distances from the original source, thereby posing a health risk to neighboring communities (Douglas et al., 2018; Dungan, 2010; Nygard et al., 2008). However, the environmental fate and transport of bioaerosols is strongly influenced by meteorological conditions, including humidity, temperature, wind velocity, and precipitation (Dungan, 2010).

A recent study conducted in an area of the Netherlands that contained regions with high livestock density modeled endotoxin and particulate matter concentrations using both land use regression and dispersion models (de Rooij et al., 2019). The study found that endotoxin exposure was significantly associated with respiratory symptoms in persons living in livestock dense areas (de Rooij, Smit, et al., 2019). Specifically, significant protective health effects (i.e., reduced prevalence of atopic sensitization and asthma) were observed with increasing endotoxin concentration at the lower range of exposure, while the prevalence of adverse health effects, such as wheeze and cough, increased significantly with increasing endotoxin concentration at the higher range of exposure (de Rooij, Smit, et al., 2019). The results suggest that bioaerosol emissions from livestock farms can have considerable effects on the health of nearby residents (de Rooij, Smit, et al., 2019). While inhalation is considered the most important exposure route (Basinas et al., 2013), deposition on fomites, food crops, and water bodies, and subsequent ingestion is also a concern (Dungan, 2010).

Cattle can excrete a number of different zoonotic pathogens that can aerosolize and infect humans through inhalation (Cambra‐López et al., 2010; McDaniel et al., 2014; Smit & Heederik, 2017). For example, *Coxiella burnetii*, which is the causative agent of Q fever in humans, is shed in high numbers in the birth products of infected cattle (McDaniel et al., 2014; Porter et al., 2011). Humans are often infected through contact with infected fluids or through the inhalation of contaminated dust (McDaniel et al., 2014; Porter et al., 2011). Generally, farmers, veterinarians, and those who live in close proximity to livestock facilities are at increased risk for airborne transmittable zoonoses (Cambra‐López et al., 2010; McDaniel et al., 2014; Porter et al., 2011; Smit & Heederik, 2017).

Antibiotic‐resistant bacteria from livestock operations (e.g., beef, dairy, and pig) can also become airborne, especially on high intensity farms or confined feed lots (Chapin et al., 2005; de Rooij et al., 2019; McEachran et al., 2015; Navajas‐Benito et al., 2017). Residential exposure to livestock‐related bacteria and AMR genes (i.e., tetW and mecA) was recently demonstrated through air measurements at residential sites in the Netherlands (de Rooij, Hoek, et al., 2019). Specifically, AMR genes were detected in bioaerosols at sites up to 1200 meters away from livestock farms (de Rooij, Hoek, et al., 2019). However, there are still knowledge gaps surrounding the potential transmission routes between dairy cattle, humans, and the environment (Burgess & French, 2017).

#### 4.1.2 Gases

Nitrogen oxides (NO_*x*_) have been associated with airway inflammation and epidemiological studies have reported an association between NO_*x*_ and bronchitis symptoms in children diagnosed with asthma (WHO, 2017). Reductions in lung function have also been observed in persons exposed to NO_*x*_ (WHO, 2017). Furthermore, NO_*x*_ plays a role in the development of ozone (O_3_), which can cause serious respiratory symptoms, trigger asthma, aggravate chronic respiratory diseases, and reduce lung function in exposed humans (Townsend et al., 2003; Von Mutius, 2000; WHO, 2017). Additionally, exposure to NO_*x*_ may worsen or lengthen the duration of certain viral infections, such as human rhinovirus (Spannhake et al., 2002; Townsend et al., 2003). Reactive nitrogen can also serve as an important driver of particulate air pollution globally (Townsend et al., 2003). NO_*x*_ emissions are typically produced by long‐term manure storage systems, fossil fuel combustion, and fertilizer use on farms and may contribute substantially to the environmental health risk associated with dairy production (Havlikova et al., 2008). VOCs also contribute to ozone formation when combined with NO_*x*_ and sunlight (Place & Mitloehner, 2010). Important sources of VOCs on dairy farms include silage and stored manure (Place & Mitloehner, 2010).

Ammonia is also a serious problem for both human and animal health and can contribute to respiratory disease, as well as decreased livestock performance, which can indirectly influence human health (Place & Mitloehner, 2010). Ammonia can also contribute to the eutrophication and acidification of aquatic ecosystems (FAO et al., 2006; Place & Mitloehner, 2010), as well as the eutrophication of terrestrial ecosystems (Havlikova et al., 2008). Important sources of ammonia on dairy farms include long‐term manure storage lagoons, fresh manure deposition, and the application of manure to agricultural fields (Place & Mitloehner, 2010; Won et al., 2017). However, there is significant variation in ammonia emissions from farm to farm depending on management practices, including cow diet, manure management system, method of manure application to agricultural fields (e.g., spraying or injection), and season (Place & Mitloehner, 2010).

### 4.2 Water Pollution

Dairy cattle, and other livestock, have a major impact on water use and availability, water quality, hydrology, and the health of aquatic ecosystems (FAO et al., 2006). For example, in the United States, livestock accounts for approximately 55% of soil erosion, 32% of nitrate loading to freshwaters, and 33% of phosphate loading to freshwaters (FAO et al., 2006). Globally, the livestock sector accounts for almost 10% of anthropogenic water use, primarily for the irrigation of feed crops (FAO et al., 2006). Livestock production may also be the single largest sectoral source of water pollution (FAO et al., 2006). Major sources of water pollution from dairy farms include animal wastes, pharmaceutical residues (e.g., antibiotics and hormones), fertilizers and pesticides used for growing feed crops, and sediment from eroded pastures (FAO et al., 2006). Antibiotic‐resistant bacteria and their genes can also act as environmental contaminants (Aitken et al., 2016; Oliver et al., 2011; Tripathi & Tripathi, 2017).

#### 4.2.1 Water Scarcity

Water scarcity is a growing issue, especially in Latin America and Sub‐Saharan Africa (FAO et al., 2006). While water use varies by type of animal, farming system characteristics, and region, the livestock production sector tends to have high water use and is contributing to water depletion trends globally (FAO et al., 2006). Livestock production requires water for animals to drink as well as water for servicing animals (e.g., to wash animals), and in the case of industrialized farms water is also needed for cleaning equipment, cooling facilities, and waste disposal (FAO et al., 2006). For dairy cattle, water requirements are estimated to range from 21.8 liters per animal per day to 127 liters per animal per day depending on the regional climate and farm production system (FAO et al., 2006). Feed production also requires water use and crops can deplete water through evapotranspiration (FAO et al., 2006).

#### 4.2.2 Biological Pollutants

Cattle also excrete a number of different zoonotic pathogens that can contaminate the environment and cause illness in humans (Cavirani, 2008; FAO et al., 2006; Ferguson et al., 2003; McDaniel et al., 2014). Contamination of water with pathogens occurs through animal contact with waterways (Collins et al., 2007; Davies‐Colley et al., 2004), through fecal runoff into surface waters (Collins et al., 2007; FAO et al., 2006), or through the leaching of fecal matter through the soil matrix into groundwater (Collins et al., 2007; Ferguson et al., 2003). Fecal runoff can increase during periods of heavy rainfall (Ferguson et al., 2003; Jokinen et al., 2012; McBride et al., 2014) and under high livestock densities (Castro‐Hermida et al., 2009; Collins et al., 2007). For example, catchment scale modeling has specifically shown high concentrations of ruminant *Campylobacter* strains during flood events as a result of agricultural runoff (McBride et al., 2011). Studies have also shown that heavy rainfall events can significantly increase surface runoff of *Cryptosporidium* oocysts over agricultural land (Davies et al., 2004; Davies‐Colley et al., 2008; Lal et al., 2013; Tryland et al., 2011). Humans can then be exposed to waterborne zoonotic pathogens through recreational contact with waterways or through the consumption of contaminated drinking water (Bridgman et al., 1995; Cavirani, 2008; Hoxie et al., 1997; McDaniel et al., 2014; Rizak & Hrudey, 2008).

Waterborne transmission of bovine zoonotic pathogens has been documented for a number of pathogens (WHO et al., 2012) and presents an important public health risk in both lower‐ and higher‐income nations. For example, an outbreak of *Escherichia coli* O157 in Swaziland cattle was thought to be the source of more than 40,000 human cases of waterborne infection (Effler et al., 2001; WHO et al., 2012). Additionally, a study that assessed the impacts of intensive dairy farming and border strip irrigation on the leaching of *Campylobacter* spp. to shallow groundwater in a catchment in New Zealand found *Campylobacter* in 12% of samples from five wells over a 3‐year period (Close et al., 2008). The probability of *Campylobacter* infection was estimated at 60–75% during the irrigation season and epidemiological assessment of the region indicated a statistically significant increase campylobacteriosis (relative risk (RR) = 1.51, 95% CI 1.33–1.72), cryptosporidiosis (RR = 5.33, 95% CI 4.12–6.90), and salmonellosis (RR = 2.05, 95% CI 1.55–2.71) rates in areas of dairy farming with major irrigation schemes compared to areas without dairy farms (Close et al., 2008). Typing of *Campylobacter* samples from environmental water also found an overlap between strains isolated from human cases and ruminants, suggesting that the consumption of untreated drinking water or recreational contact with surface water contaminated by livestock was an important source of infection in rural areas (French et al., 2011).

Antibiotic‐resistant bacteria and their genes can also contaminate the environment (Aitken et al., 2016; Oliver et al., 2011; Tripathi & Tripathi, 2017) and have been isolated from dairy wastewater; soil from dairy farms; dairy manure; and the dairy farm environment (Collis et al., 2019; Noyes et al., 2016; Oliver et al., 2011; Pitta et al., 2016). Antibiotic‐resistant bacteria have also been detected in surface water, groundwater, sediments, and wetlands (Aitken et al., 2016; Oliver et al., 2011; Tripathi & Tripathi, 2017). Freshwater ecosystems support the distribution and evolution of AMR because they are sites that facilitate genetic exchange through horizontal gene transfer (Tripathi & Tripathi, 2017).

#### 4.2.3 Chemical Pollutants

Cattle manure and urine, as well as farm wastewater, can contain high levels of nutrients, drug residues, pathogens, or heavy metals that can enter waterways or accumulate in soils (FAO et al., 2006; Won et al., 2017). These pollutants can enter waterways either directly from runoff from farm buildings, spills or the failure of manure storage facilities, the deposition of fecal matter directly to streams, transport through soil layers via drainage waters on farms, or contamination can occur indirectly from surface runoff and overland flow from pastures or agricultural fields (FAO et al., 2006). Livestock can also contribute to soil compaction, which can in turn reduce water infiltration, increase overland runoff, and lower groundwater tables (FAO et al., 2006), potentially contributing to both water scarcity and water quality challenges.

Nitrogen and phosphorus are critical pollutants from dairy farms to surface waters, groundwater, and marine waters (OECD, 2004). When nitrogenous fertilizers are applied to crops, only a portion is taken up by plants and the rest is often transported downstream or downwind (FAO et al., 2006). Manure and urine are also important sources of nitrogen emissions on dairy farms (FAO et al., 2006). Excessive nitrate can pollute the environment and is a direct threat to human health (FAO et al., 2006; OECD, 2004). High levels of nitrate in drinking water can lead to the development of methemoglobinemia in infants (FAO et al., 2006; Gupta et al., 1999). Nitrate toxicity has also been linked to abortions in pregnant women and certain forms of cancer in adults (FAO et al., 2006; Johnson et al., 2010; Townsend et al., 2003). Specifically, elevated nitrate levels in water lead to the formation of potentially carcinogenic N‐nitrosamines (van Maanen et al., 1996). In some studies, the long‐term consumption of nitrate in drinking water has been positively associated with a higher risk for non‐Hodgkin's lymphoma, stomach, colorectal, bladder, breast, and ovarian cancers, and thyroid disease (Espejo‐Herrera et al., 2016; Fachiroh et al., 2017; Gulis et al., 2002; Inoue‐Choi et al., 2013; Jones et al., 2016; Schullehner et al., 2018; Ward et al., 2018; Weyer et al., 2001), although findings over time have not been consistent. Phosphorus is not directly toxic to human beings, but it is often the limiting nutrient in aquatic ecosystems (FAO et al., 2006) and changes in concentration can severely alter ecosystem functions.

### 4.3 Environmental Health Impacts Summary

Overall, dairy farming contributes substantially to air pollution and water pollution. Specifically, livestock production contributes to local and regional air pollution (FAO et al., 2006). Particulate matter, ammonia, nitrogen oxides, and VOCs are major air pollutants that are emitted on dairy farms (FAO et al., 2006; Havlikova et al., 2008; Place & Mitloehner, 2010; Won et al., 2017).

Important water pollutants from dairy farms include fertilizers, nutrients, zoonotic pathogens, pesticides, sediment, antibiotics, hormones, and other drug residues. It is difficult to compare the contribution to water pollution across livestock sectors, although cattle, due to their large size, excrete substantial quantities of manure, often containing high levels of nutrients (Sheldrick et al., 2003). Furthermore, a recent study estimated manure production per day per 100 kg of live animal mass, using average adult animal body mass, and reported that dairy cattle produced more manure than any other livestock species (Vermeulen et al., 2017). However, stocking densities will inevitably influence the contribution of different livestock in a given area.

Total global livestock excreta have been estimated to contain 94 million tons of nitrogen, 21 million tons of phosphorus, and 67 million tons of potassium, and cattle were the largest contributors to the total (60%), while pigs and poultry only contributed 10% and 9%, respectively (Sheldrick et al., 2003). Livestock production has steadily increased over time (Thornton, 2010) and, logically, livestock excreta and nutrient quantities have increased along with livestock numbers.

Manure is a particularly important source of environmental pollution on dairy farms and management of manure in a way that minimizes environmental health risks presents a serious challenge. The accumulation of manure on dairy farms contributes to the emission of ammonia (FAO et al., 2006; Havlikova et al., 2008; Place & Mitloehner, 2010; Won et al., 2017), and the environmental dispersal of zoonotic pathogens (Cavirani, 2008; McDaniel et al., 2014; Toth et al., 2013). Furthermore, manure contains excess nutrients that can end up in soils and streams, causing eutrophication, algal blooms, reduced light penetration, and decreased oxygen availability in aquatic ecosystems (FAO et al., 2006; OECD, 2004; Won et al., 2017).

## 5 Ecosystem Health Impacts

The causal links between environmental changes and human health are often indirect and complex (Corvalan et al., 2005; Ingram, 2012). The impacts of environmental change can be displaced in time and space and may be dependent upon a number of different modifying forces (Aron & Patz, 2001; Corvalan et al., 2005). Generally, wealthier individuals and groups have the ability to distance themselves spatially and temporally from the ecological consequences of their consumption choices and the effects are often shifted to resource‐poor populations that are more vulnerable to the adverse consequences (Aron & Patz, 2001; Corvalan et al., 2005). While the links between environmental change and public health are therefore clearest in poor communities, wealthy communities cannot completely avoid the adverse effects of environmental degradation (Corvalan et al., 2005). For example, climate change can stress agricultural production, often in different regions of the world from where the emissions originated, which can in turn lead to malnutrition, increased susceptibility to infectious disease, and other health issues. Critical indirect ecosystem health risks associated with dairy production include the loss of ecosystem services (i.e., the benefits obtained from the natural environment and properly functioning ecosystems), climate change, and biodiversity loss.

### 5.1 Loss of Ecosystem Services

Ecosystem services can be defined as “the conditions and processes through which ecosystems, and their biodiversity, sustain and fulfil … life (Ingram, 2012, pg. 232),” and they are classified into provisioning services, such as food, water, materials, and fuels; regulating services, including climate and flood control, water purification, and disease regulation; supporting services, such as nutrient cycling and crop pollination; and cultural services that provide nonmaterial benefits, including cultural, spiritual, educational, esthetic, and recreational experiences (Falkenmark et al., 2007; Ingram, 2012; Reid et al., 2005). Below, a number of links between agricultural development, the loss of ecosystem services, and human health and wellbeing are briefly reviewed. Links are presented by categories of ecosystem services: provisioning, regulating, supporting, and cultural.

#### 5.1.1 Provisioning Services

##### 5.1.1.1 Provision of Food

Agricultural development can benefit human health by increasing food availability and security, and improving overall nutrition, particularly in lower‐income nations (Johnson et al., 2010; Sanchez & Swaminathan, 2005; Townsend et al., 2003). Dairy cattle can provide milk and dairy products that are an important source of protein, vitamins and minerals (Hess et al., 2016; Kliem & Givens, 2011; Pereira, 2014). Dairy cattle are also an important source of meat (FAO, 2017). However, the production of animal‐based foods, including dairy products, can contribute to food distribution inequality and unbalanced diets (Townsend et al., 2003). The global distribution of food is uneven and the world faces the double burden of malnutrition, in which undernutrition coexists along with overweight or obesity‐related health concerns (WHO, 2018c). Undernutrition and hunger are now primarily caused by inequitable food distribution, rather than by inadequate global production (Smil, 2000; Townsend et al., 2003). This is in part due to the fact that in many regions with intensifying agricultural systems, many of the crops produced, typically using large quantities of nitrogenous fertilizers, are used as supplemental livestock feeds (FAO et al., 2006; Townsend et al., 2003). For example, many intensive dairy farms rely on supplemental feeds, such as maize in the United States (FAO et al., 2006) and palm kernel expeller (PKE) in New Zealand (Foote et al., 2015), in addition to, or in place of, grazing.

The trend in the use of grain crops for animal feed can lead to nitrogen losses to the environment (Townsend et al., 2003). Globally, 33% of arable land is used for feed crops (FAO et al., 2006). However, the proportion of global arable land used specifically for feed crops for dairy production is unknown, although some regional estimates of feed crop use by different livestock sectors have been made. For example, in the European Union more arable land is allocated to feed crops for dairy production than for beef, pork, or poultry production systems (Lesschen et al., 2011) and in 2010 dairy cattle used 29% of total feed by dry mass, while other cattle used 34%, pigs 17%, chickens 9%, sheep and goats 8%, and other animals 3% (Hou et al., 2016). However, substantial differences in estimated feed use, as well as vast differences in estimated nitrogen losses, between countries were reported (Hou et al., 2016). Additionally, each livestock production system uses different feed inputs which affect the estimated share of feed trade per sector and the geographical patterns of nitrogen losses (Chatzimpiros & Barles, 2013). Therefore, generalizations about feed use by different livestock sectors should be made with caution.

Due to uncertainty surrounding feed crop use, it is difficult to estimate the proportion of total nitrogen losses from feed crops specifically attributable to dairy production as opposed to other livestock sectors. However, several studies have attempted to estimate nitrogen losses from feed crops by livestock sector at the national level. For example, a study conducted in France estimated that dairy production in France used 2.3 kg of nitrogen per capita per year (kg N/cap/year), of which 48% was nitrogen that was not recovered in animal biomass or exported as manure or slaughter waste (Chatzimpiros & Barles, 2013). Only 11% was recovered in retail dairy products and approximately 35% was returned to agricultural production as manure (Chatzimpiros & Barles, 2013). Beef and pork production used 11.1 and 7.5 kg N/cap/year, respectively, of which 35% and 53% was nitrogen that was not recovered in retail products or exported as manure or slaughter waste (Chatzimpiros & Barles, 2013). Overall, crop cultivation for livestock feed was the primary cause of total nitrogen loss for dairy, beef, and pork production, contributing to more than 75% of losses for each sector (Chatzimpiros & Barles, 2013). These estimates were comparable to nitrogen loss factors calculated for U.S. livestock production systems (Leach et al., 2012) and similar nitrogen losses may be expected in other temperate countries with industrialized agricultural sectors. However, nitrogen losses may exhibit considerable geographic variation due to different farm management practices and nitrogen use efficiencies.

Nitrogen losses can result in the degradation of both aquatic and terrestrial ecosystems, which can in turn lead to food supply losses (Townsend et al., 2003). For example, the loss of reactive nitrogen to waterways can drive the eutrophication of marine coastal waters, contribute to harmful algal blooms and fish kills, and cause environmental degradation that can harm shellfish and fisheries (Townsend et al., 2003). Nitrogen losses to the atmosphere can also lead to high tropospheric ozone levels and may cause extensive crop damage (Chameides & Kasibhatla, 1994; Townsend et al., 2003). Diet deficiencies from loss of provisioning services can then lead to physical and developmental problems in children (Corvalan et al., 2005).

##### 5.1.1.2 Provision of Freshwater

Numerous aspects of the hydrological cycle are regulated by the natural functions of ecosystems (Corvalan et al., 2005). Freshwater is essential for human health and agricultural development, including dairy farming, and can influence or interfere with the hydrological cycle in a variety of ways (Corvalan et al., 2005;Falkenmark et al., 2007 ; FAO et al., 2006). Irrigated agriculture and livestock production require increasing volumes of water (Corvalan et al., 2005; Falkenmark et al., 2007; FAO et al., 2006). For example, dairy production requires substantial quantities of freshwater, both for drinking and for servicing (FAO et al., 2006). Lactating cows require considerably more drinking water each day than goats, sheep, camel, chickens, or swine (FAO et al., 2006). Additionally, only industrial swine production requires more water per animal per day for servicing than industrial dairy production (FAO et al., 2006). However, dairy cattle that are grazed extensively require less than a quarter of the water for servicing that dairy cattle in industrial systems require (FAO et al., 2006). Water withdrawals or diversions may reduce the availability of water, not just for use in agricultural systems, but also for communities and natural ecosystems downstream (FAO, 2003; Wu, 2008).

#### 5.1.2 Regulating Services

##### 5.1.2.1 Regulation of Infectious Disease

Patterns of infectious disease transmission are often influenced by environmental or climatic factors, which can alter the spread of infectious agents between humans, the dissemination of agents, as well as the activity of vector organisms (Corvalan et al., 2005). The anthropogenic alteration of ecosystems and environmental conditions can change the natural influence of environmental or climatic factors on the range and activity of infectious agents (Corvalan et al., 2005). However, the direction and extent of change in the incidence of a given infectious disease can vary considerably depending upon the type of ecosystem affected, the type of land use change, and disease‐specific transmission dynamics, as well as sociocultural changes and the vulnerability of human populations (Corvalan et al., 2005). Not all ecosystem change will lead to an increase in infectious disease incidence; ecosystems can be a source of infectious agents and the alteration of an ecosystem can, in some cases, reduce the incidence of disease in an area (Patz et al., 2005). Ecosystem modification has frequently been used as a tool to control disease vectors (Patz et al., 2005). Nevertheless, on balance, the current scale of ecosystem alteration may lead to the emergence or reemergence of infectious diseases (Patz et al., 2005).

Together, land use change and global climate change can significantly alter temperature, precipitation, biogeochemical cycles, nutrient concentrations, water chemistry, and exposure to sunlight (Myers & Patz, 2009). These ecosystem alterations can then influence pathogen, vector, or host density, genetics, life cycles, or exposure pathways (Myers & Patz, 2009). Infectious disease risks are also strongly affected by destruction or encroachment into wildlife habitat; changes in the distribution and availability of surface waters (e.g., through irrigation systems and stream diversions); and agricultural land use changes, including the proliferation of livestock and crops (Corvalan et al., 2005). Habitat alteration can be a particularly important driver of the emergence or reemergence of vector‐borne or zoonotic diseases, as it can result in changes to vector breeding sites or to reservoir host spatial distribution (Corvalan et al., 2005; Patz et al., 2008).

Agricultural land use change can also increase connectivity with certain wildlife species, both between humans and wildlife and between domestic animals and wildlife. This increased interconnectivity can influence the emergence and spread of zoonotic pathogens (Corvalan et al., 2005). Additionally, the genetic resistance of vectors and pathogens to pesticides and antimicrobials used in agricultural systems can drive the emergence of infectious disease (Corvalan et al., 2005). However, agricultural development and land use change does not always lead to an increase in the incidence of infectious diseases. A systematic review of anthropogenic land use changes and infectious diseases that included 305 articles, most of which were observational studies, found that 59.3% of studies documented an increase in pathogen transmission associated with land use change, while 30.4% of studies found a variable response, 10.4% saw a decrease, and 2.4% documented no change in pathogen transmission related to land use change (Gottdenker et al., 2014). Generally, the diseases that are most sensitive to environmental factors such as land use change and climate change are those that are indirectly transmitted (e.g., waterborne or foodborne diseases) or those that have an intermediate host or vector as part of their life cycle (Patz et al., 2008).

##### 5.1.2.2 Climate Regulation

Case studies suggest that land use change and environmental degradation have reduced the capacity of certain ecosystems to buffer against extreme climatic conditions (Corvalan et al., 2005). All ecosystem services are sensitive to climatic conditions and will be affected by anthropogenic climate change (Corvalan et al., 2005). Therefore, as ecosystems lose the capacity to buffer against extreme conditions, additional ecosystem services will be lost, which will in turn undermine human health and wellbeing. The contribution of the dairy sector to global climate change and the potential human health impacts associated with climate change are discussed in further detail in section 5.2.

#### 5.1.3 Supporting Services

##### 5.1.3.1 Nutrient Management

Ecosystems play a key role in the cycling and distribution of nutrients (Corvalan et al., 2005). Nutrient cycling is a fundamental service that underpins the life and health of organisms on Earth (Corvalan et al., 2005). The production and improper management of manure and waste from livestock systems, including dairy production systems, can lead to excessive nutrient leaching (FAO et al., 2006; OECD, 2004). Additionally, the application of nitrogenous and phosphatic fertilizers in agricultural systems around the world has increased substantially in recent decades (Falkenmark et al., 2007; FAO et al., 2006). The flux of reactive nitrogen to the oceans increased by approximately 80% from 1860 to 1990, and the application of phosphorus has increased threefold since 1960 (Falkenmark et al., 2007). Agricultural nutrient and waste management can both directly and indirectly harm human health (Townsend et al., 2003), although it is difficult to determine the extent to which dairy production is responsible for health hazards as compared with other livestock sectors. The potential indirect health effects associated with nutrient loading are briefly reviewed here.

The disruption of nutrient cycling is closely linked to the loss of provisioning services. Disruptions in nutrient cycles can decrease soil fertility, reduce crop yields, and impair household nutritional status (Corvalan et al., 2005). Excess nutrient loading can also stimulate the eutrophication of aquatic ecosystems and may be indirectly harmful to human health (FAO et al., 2006; Johnson et al., 2010; OECD, 2004; Townsend et al., 2003). Eutrophication can lead to the depletion of oxygen in waters, shifts in wildlife habitat characteristics, and changes in species distribution and composition (FAO et al., 2006; Won et al., 2017). Nutrient concentration and eutrophication have been linked to an increase in harmful algal blooms (Anderson et al., 2008; Burkholder, 1998; Gobler et al., 2012; Heisler et al., 2008) and to cholera outbreaks (Colwell & Huq, 2001; Cottingham et al., 2003; Epstein, 1993; Johnson et al., 2010). The degradation of aquatic ecosystems can in turn reduce the availability and safety of fish and shellfish for human consumption (Townsend et al., 2003).

Sustained increases in nutrient loading of ecosystems from agricultural development, including dairy production, and other human activities is contributing to environmental deterioration and creating human health risks (Corvalan et al., 2005). Excess nutrient loading may indirectly contribute to increases in both noncommunicable and communicable diseases (Johnson et al., 2010; Townsend et al., 2003).

There are a number of different ways that excess nitrogen or phosphorus availability in the air and water, due to agricultural activities, can contribute to the incidence of respiratory diseases, cardiovascular disease, and certain cancers (section 4.1.2; Townsend et al., 2003; Johnson et al., 2010). Additionally, excess soil nitrogen can stimulate higher pollen production in some plant species, such as ragweed, and can therefore contribute to an increase in human allergenic responses to pollen (Townsend et al., 2003).

Environmental nitrogen and phosphorus loading can also influence the abundance and distribution of infectious disease vectors, like mosquitoes (Johnson et al., 2010; Townsend et al., 2003). Nutrient availability can influence vectors through the modification of their habitat or food sources (Townsend et al., 2003). For example, some studies have found positive correlations between inorganic nitrogen levels and larval abundance for mosquitos that are carriers for malaria (Rejmánkova et al., 1991; Teng et al., 1998), La Crosse encephalitis, Japanese encephalitis, and West Nile Virus (Sunish & Reuben, 2001; Townsend et al., 2003; Walker et al., 1991). Nutrient enrichment also plays a complex role in the emergence or reemergence of infectious disease in both humans and animals. Specifically, environmental nutrient enrichment can increase pathogen and parasite abundance (Johnson et al., 2010; Lafferty, 1997; Lafferty & Holt, 2003; McKenzie & Townsend, 2007).

#### 5.1.4 Cultural Services

There are a number of nonmaterial benefits that individuals and communities can gain from ecosystems (Corvalan et al., 2005; Sandifer et al., 2015). Healthy ecosystems provide space for recreation, physical activity, tourism, esthetic appreciation, inspiration, and educational opportunities (Corvalan et al., 2005; Sandifer et al., 2015). These services can boost mental and physical health and enhance social and cultural ties (Corvalan et al., 2005; Sandifer et al., 2015). While these benefits may be difficult to quantify, evidence suggests that people and communities highly value the cultural services that ecosystems provide (Corvalan et al., 2005; Pröbstl‐Haider, 2015). Therefore, the loss of natural resources and ecosystem cultural services may have negative consequences for human health and wellbeing (Corvalan et al., 2005).

### 5.2 Climate Change

Important greenhouse gas (GHG) emissions associated with dairy farming include methane (CH_4_), nitrous oxide (N_2_O), and carbon dioxide (CO_2_) (FAO, 2010; FAO et al., 2006; Grossi et al., 2018; OECD, 2004; Place & Mitloehner, 2010; Won et al., 2017). Modern farms emit CO_2_ largely from fossil fuel combustion for on‐farm processes, transportation, and electricity generation (FAO, 2010; Place & Mitloehner, 2010). Livestock production also contributes indirectly to CO_2_ emissions through the burning of fossil fuels to produce fertilizers, land use change for feed production and grazing, and land degradation (FAO, 2010; FAO et al., 2006). However, the dairy sector's contribution to climate change is dominated by CH_4_ and N_2_O from enteric fermentation and manure management, including the anaerobic decomposition of manure, application or deposition of manure, and indirect manure emissions (FAO et al., 2006; Grossi et al., 2018).

The 100‐year global warming potential (i.e., the ability of a GHG to trap extra heat in the atmosphere over time relative to CO_2_) of CH_4_ and N_2_O are more than 20 and 300 times greater than that of CO_2_, respectively (Grossi et al., 2018; IPCC, 2007; Place & Mitloehner, 2010). The majority of CH_4_ emitted from dairy farms is produced through enteric fermentation in cattle, although manure can also be a source of emissions (FAO, 2010; Grossi et al., 2018; Place & Mitloehner, 2010; Won et al., 2017). N_2_O is formed during the microbially facilitated process of denitrification, the natural cycle by which nitrate is reduced to nitrogen gas, and major sources on dairy farms include long‐term manure storage lagoons, nitrogenous fertilizers, and manure spread on agricultural fields (FAO, 2010; Grossi et al., 2018; Place & Mitloehner, 2010).

**Figure 2:**
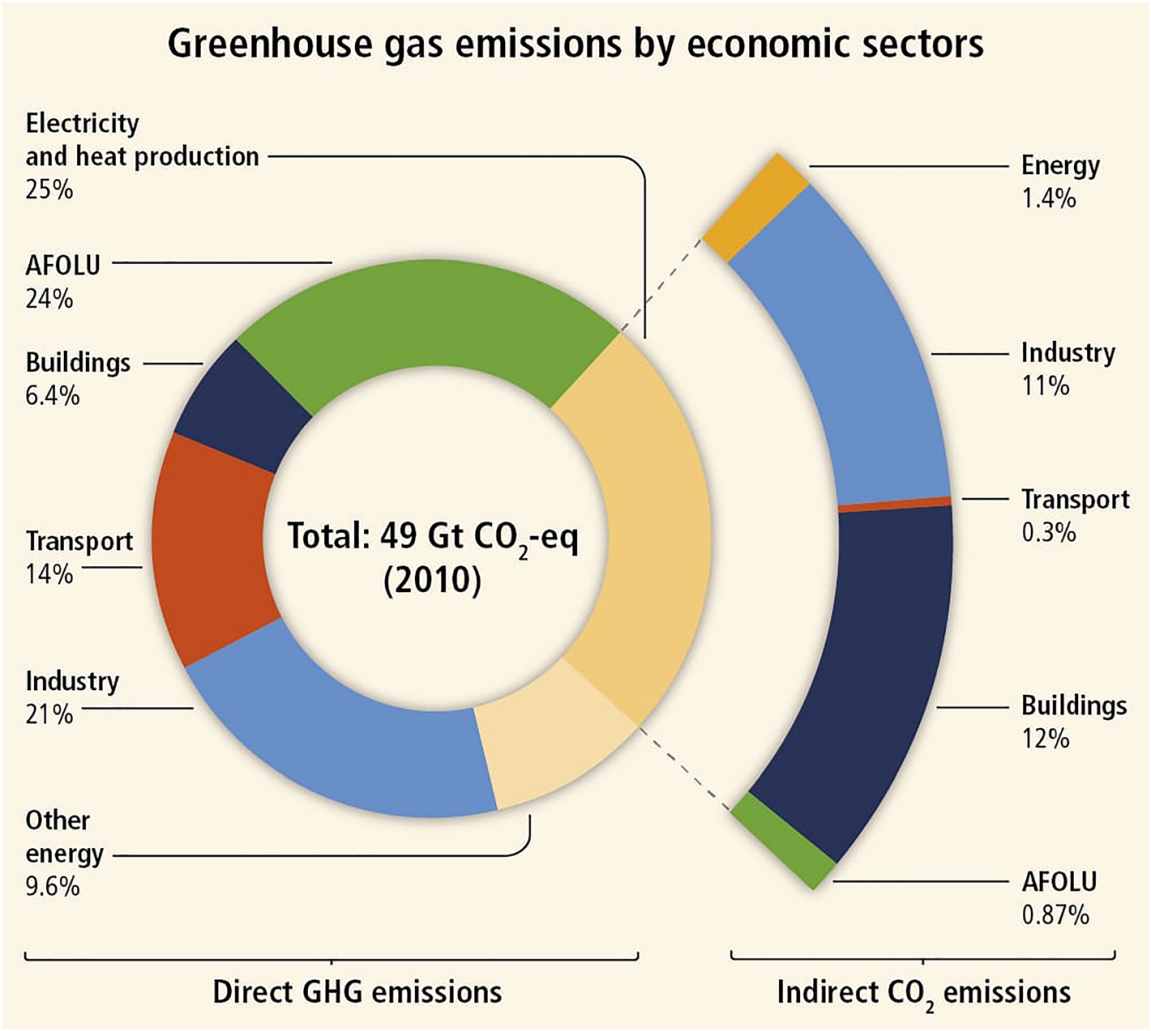
Global anthropogenic GHG emissions by economic sectors (IPCC, 2014, pg. 47).

The Food and Agriculture Organization of the United Nations (FAO) estimated that in 2007 the global dairy sector emitted 1,969 million tons of CO_2_ equivalents (±26%), of which 1,328 million tons were attributable to milk production, 151 million tons were attributable to meat from culled cattle, and 490 million tons were attributable to meat from fattened calves (FAO, 2010). This estimate does not include the emissions related to land use change under constant management practices, capital goods (i.e., farm equipment and buildings), on‐farm milking or cooling, or retail activities (e.g., refrigeration and disposal of packaging) (FAO, 2010). In total, the global dairy sector contributes approximately 4% to total anthropogenic GHG emissions (FAO, 2010). However, the contribution of the dairy sector to global GHG emissions is relatively small compared to energy, industrial processes, and transport, especially as emissions from the dairy sector represent only a portion of the total emissions from agriculture, forestry and other land uses (AFOLU; Figure 2; IPCC, 2014).

Overall, cattle, as large ruminants, contribute substantially more to climate change than other smaller, nonruminant livestock species, such as poultry and pigs (Caro et al., 2014). Specifically, from 1961 to 2010, beef cattle contributed approximately 54% of total livestock emissions, followed by dairy cattle (17%), sheep (9%), buffalo (7%), pigs (5%), and goats (4%) (Caro et al., 2014).

The combined effects of land use change and climate change, and in particular extreme climate events, on human health can be severe, both in terms of direct health outcomes, such as morbidity and mortality from heatwaves, floods, droughts, and storms (Corvalan et al., 2005; IPCC, 2014), and in terms of environmentally mediated diseases (Foley et al., 2005; Smith et al., 2014). There are also a number of indirect health effects associated with climate change that arise due to social, economic, and political disruptions (McMichael et al., 2006). Direct and indirect health impacts associated with climate change have been extensively documented in the literature and are not outlined further in this review.

### 5.3 Loss of Biodiversity

The FAO has stated that the livestock sector may be the leading driver of biodiversity loss (FAO et al., 2006). However, it is difficult to quantify the contribution of the livestock sector because biodiversity loss is typically caused by a combination of different processes of environmental degradation (FAO et al., 2006). Additionally, the type of livestock production system strongly influences the type and extent of threat to biodiversity (FAO et al., 2006). Intensive livestock production systems, which seek to increase yields per unit area, typically through increased stocking rates and the use of off‐farm inputs, have contributed to biodiversity loss through pollution (FAO et al., 2006). Extensive systems, which seek to increase yields through the expansion of farmland area, have caused dramatic biodiversity losses due to forest fragmentation or deforestation and the introduction of invasive plant species (FAO et al., 2006). Dairy farms can be either intensive or extensive and the threats to biodiversity differ by the type of production system (Table 1).

**Table 1:** Expert Ranking of Livestock‐Related Threats to Biodiversity Resulting From the Different Mechanisms and Types of Production Systems (FAO et al., 2006)

Mechanism of livestock sector induced biodiversity loss	Type of livestock production system	
	Extensive production	Intensive production
Forest fragmentation	**↗**	**↑**
Land use intensification	**↗**	**↑**
Desertification	**→**	
Forest transition (reversion of former pastures)	**↗**	
Climate change	**↗**	**↑**
Invasive livestock	**↘**	
Plant invasions	**↘**	**→**
Competition with wildlife	**↘**	**↑**
Overfishing		**↗**
Livestock diversity erosion		**↑**
Toxicity		**↑**
Habitat pollution	**→**	**↑**

*Note*. Relative level and type of threat to biodiversity resulting from the different mechanisms. “Extensive” and “Intensive” refer to the importance of the contributions from both sides of the continuum of livestock production systems. Red shading indicates the level of past impact: dark red = very strong; red = strong; dark pink = moderate; light pink = weak; white = no effect. Arrows indicate the direction of current trends: ↘ = decreasing; → = stable; ↗ = increasing; ↑ = rapidly increasing.

However, livestock can also positively influence biodiversity (FAO et al., 2006). For example, in Europe traditional grazing creates and maintains sward structural heterogeneity, which is seen as positively affecting biodiversity (FAO et al., 2006). Additionally, livestock can condition the soil through the deposition of manure and urine, which can improve soil fertility and support plant life (FAO et al., 2006;Hart, 1999 ; Horrigan et al., 2002). Livestock can also support plant propagule dispersal (FAO et al., 2006). However, when traditional pastures become more intensively managed, many of these benefits are lost, along with much of the remaining biodiversity (FAO et al., 2006).

While there is limited research regarding the influence of dairy production on biodiversity, a study in New Zealand that investigated plant distribution and soil invertebrates from a site before and after conversion to intensive dairy farming reported that only 31% of the original 65 native vascular plant species were found after conversion to dairy farming and 27 exotic species had been introduced since the original survey in 1970 (Bowie et al., 2016). There were notable declines in native earthworm populations, but there were no indications of soil invertebrate biodiversity loss (Bowie et al., 2016). However, a study in Ireland reported that total arthropod abundance was significantly greater in more intensively managed swards of dairy farms and there was greater breeding bird species richness and abundance along field boundaries on dairy farms compared with nondairy farms (McMahon et al., 2010). The influence of dairy farming on biodiversity may vary by site and species involved.

Moreover, biodiversity loss, like many other environmental impacts, can be displaced in time and space. For example, farm imports, such as supplemental feeds and fertilizers, can have substantial impacts on biodiversity in the location in which they are produced, but not necessarily in the locations where they are used. PKE is produced as a by‐product of the palm oil industry in Southeast Asia and is exported and sold as a supplemental feed for livestock. New Zealand is the largest global importer of PKE and it is used almost exclusively as a supplemental feed for dairy production (Foote et al., 2015). While the use of PKE does not seem to pose a substantive risk to local biodiversity in New Zealand, oil palm cultivation is responsible for 11 million hectares of deforestation globally (Nicholas et al., 2018). Such deforestation greatly reduces terrestrial biodiversity and a study in Myanmar reported that at certain sites of oil palm cultivation, local low‐income populations experienced reduced dietary and nutritional diversity and negative health outcomes as a result of industrial monocropping (Nicholas et al., 2018).

The relationship between human health and biodiversity is complex with many linkages (WHO & Secretariat of the Convention on Biological Diversity, 2015). Human health and wellbeing are reliant on a number of different ecosystem services that are supported by biodiversity (Sandifer et al., 2015). Specifically, biodiversity loss can harm human health through impacts on water and air quality, nutrition, noncommunicable diseases, infectious diseases, and biomedical advancements (WHO & Secretariat of the Convention on Biological Diversity, 2015). Reduced opportunities to develop new drugs, such as antibiotics, from natural products may be the most direct link between biodiversity loss and human health (WHO & Secretariat of the Convention on Biological Diversity, 2015). Additionally, natural biodiversity and biodiversity‐supported ecosystem services can provide psychological, esthetic, cultural, recreational, socioeconomic, and spiritual benefits (Sandifer et al., 2015).

Biodiversity is also the basis for global agricultural production (WHO & Secretariat of the Convention on Biological Diversity, 2015). The loss of biodiversity may reduce the availability of nutritious food sources, which may lead to serious human health consequences in certain regions of the world (World Resources Institute, 2005). Additionally, the loss of biodiversity in agroecosystems increases the vulnerability and decreases the sustainability of many food production systems (WHO & Secretariat of the Convention on Biological Diversity, 2015). Overall, the diversity of species, varieties, and breeds in agricultural production underpins human dietary diversity, nutrition, and health (WHO & Secretariat of the Convention on Biological Diversity, 2015).

Biodiversity benefits extend beyond the provision of food and other raw materials. Exposure to wild greenspace that is undeveloped by humans has been associated with a wide array of positive health and wellbeing benefits, including improvements in general health, stress reduction, increased physical activity, and reduced incidence of cardiovascular, intestinal, and respiratory diseases (Sandifer et al., 2015). While the evidence for direct linkages between health outcomes and human exposure to biodiversity are limited, there is mounting evidence that exposure to diverse natural habitats and multiple different species may be beneficial to human health (Sandifer et al., 2015). For example, a study examining the perceptions of urban greenspace users in the United Kingdom found that psychological and physical benefits associated with exposure to nature increased with species richness and habitat diversity (Fuller et al., 2007).

There is stronger evidence for a causal relationship between human exposure to natural biodiversity and the maintenance of a healthy immune system and the reduction of inflammatory diseases (Sandifer et al., 2015). Human exposure to diverse natural habitats and diverse microbial populations is considered critical for the development of normal human immune responses to allergens and disease‐causing agents (Sandifer et al., 2015; WHO & Secretariat of the Convention on Biological Diversity, 2015). Several studies have suggested that exposure to the natural environment and vegetation diversity may be protective of asthma (Donovan et al., 2018; Lovasi et al., 2008; Sbihi et al., 2015). Reduced contact with the natural environment and biodiversity, as well as biodiversity loss, leads to declining diversity of human microbiota, which can lead to immune dysfunction and disease (Sandifer et al., 2015; WHO & Secretariat of the Convention on Biological Diversity, 2015). Specifically, rapid declines in biodiversity may contribute to the increasing prevalence of allergies, asthma, and chronic inflammatory diseases in urban populations (Sandifer et al., 2015). Biodiversity loss may also influence human health through ecosystem regulation of infectious diseases (WHO & Secretariat of the Convention on Biological Diversity, 2015; World Resources Institute, 2005). While areas with higher biodiversity may support a greater diversity of pathogens, higher biodiversity may also prevent or reduce human exposure to infectious agents (WHO & Secretariat of the Convention on Biological Diversity, 2015; World Resources Institute, 2005).

### 5.4 Soil Degradation

Livestock production is the largest anthropogenic use of land and the total area occupied by grazing totals more than a quarter of the ice‐free terrestrial surface of the Earth (FAO et al., 2006). Globally, the livestock sector has caused substantial degradation of land and soils (FAO et al., 2006). Consequences of the misuse of soil resources include accelerated erosion, desertification, salinization, acidification, compaction, biodiversity loss, nutrient depletion, and loss of soil organic matter (Sanderman et al., 2017). Furthermore, soil health is both directly and indirectly linked to human health through food production and security, food nutritional quality, food safety, soil and water interactions, water filtration, environmental exposure to chemicals and pathogens, and airborne dust formation (Brevik & Sauer, 2015; Nieder et al., 2018).

Humans rely on fertile soils to feed an ever‐growing population, but soil erosion is occurring faster than soil replenishment, reducing soil fertility (Horrigan et al., 2002). It takes between 20 to 1,000 years for a centimeter of soil to form, but wind and water alone erode at least 1% of topsoil globally each year (Horrigan et al., 2002). Industrial agriculture poses a serious threat to soil health through erosion and overgrazing (Horrigan et al., 2002). The use of heavy machinery can compact soil, disrupting the soil food web, causing declines in beneficial soil organisms, and altering soil structure (Horrigan et al., 2002). Feedlot cattle and other forms of industrial animal agriculture can also contribute to the destruction of topsoil because growing grain for the industry requires substantial land (Horrigan et al., 2002). Additionally, when cattle are heavily grazed they can cause soil erosion, pugging, and compaction (Horrigan et al., 2002).

However, livestock production can also positively affect soil health. Free‐ranging cattle, when grazed sustainably, can support biodiversity (Horrigan et al., 2002). The U.S. Department of Agriculture's Agricultural Research Service found that moderately grazed land (one cow per 6.5 hectares) had higher biodiversity than both ungrazed and heavily grazed lands (Hart, 1999; Horrigan et al., 2002). Additionally, livestock have often played an important role in the recycling of nutrients and the conditioning of soil through the deposition of manure (Horrigan et al., 2002). However, industrial animal production systems tend to output manure at a higher rate than local croplands can absorb it (Horrigan et al., 2002).

### 5.5 Ecosystem Health Impacts Summary

Dairy farming contributes substantially to climate change. Enteric fermentation is an important source of methane globally and manure storage and spread and fertilizer use are important sources of nitrous oxide. Cattle, as large ruminants, contribute more to climate change than smaller, nonruminant livestock species, such as poultry and pigs (Caro et al., 2014). It has been estimated that in total, the global dairy sector contributes approximately 4% to total anthropogenic GHG emissions (FAO, 2010). However, the contribution of the dairy sector to global GHG emissions is relatively small compared to other economic sectors (IPCC, 2014). Dairy production also contributes to the loss of ecosystem services, biodiversity loss, and soil degradation, although it is difficult to quantify these impacts.

## 6 Foodborne Hazards

Food safety hazards in the dairy supply chain may be biological, chemical, or physical in nature (Smith, 2016; van Asselt et al., 2017). A comprehensive review of hazards in the dairy supply chain in Europe found that microbiological hazards are of particular concern, with chemical and physical hazards less frequently encountered in dairy products (van Asselt et al., 2017). These hazards can potentially be introduced at different phases of dairy production, including animal feed production, raw milk production on the farm, and processing either on the farm or at another off‐farm facility (Oliver et al., 2005; Smith, 2016; van Asselt et al., 2017; Zastempowska et al., 2016). While pasteurization was introduced as a method of controlling foodborne pathogens in milk and has been successful in reducing the incidence of diseases such as bovine tuberculosis and brucellosis, the consumption of unpasteurized dairy products, or products contaminated after pasteurization, continues to pose a threat to public health (LeJeune & Rajala‐Schultz, 2009; Thoen et al., 2006; van Asselt et al., 2017). While there are a number of different potential contaminants that are perceived to pose a risk to public health, the evidence for the real risk to public health varies by contaminant. There is substantial evidence that certain biological contaminants pose a real risk to public health, but for many chemical and physical contaminants there is not sufficient evidence to determine the true public health burden.

### 6.1 Biological Contaminants

While many pathogens are transmissible to humans through a number of different exposure pathways, this section will focus on pathogens commonly of concern in milk and dairy products. Foodborne outbreaks have been traced to both raw and pasteurized milk and dairy products (Oliver et al., 2005). Milk can serve as a good growth medium for certain microbes due to its high water content and neutral pH (Zastempowska et al., 2016) and pasteurization or inadequate pasteurization may not inactivate all pathogens (Oliver et al., 2005). Additionally, dairy products that have been pasteurized can still become contaminated during further processing, storage, or preparation before consumption (Oliver et al., 2005).

The presence of pathogens in milk and dairy products on farms may be due to direct contact with contaminated sources in the dairy farm environment or to excretions from cattle with either systemic disease or localized infections (e.g., mastitis) (Oliver et al., 2005; Zastempowska et al., 2016). Some foodborne pathogens can cause clinical or subclinical mastitis in cattle, in which case the pathogen can be directly excreted in milk (Oliver et al., 2005; Zastempowska et al., 2016). However, most foodborne pathogens are introduced through fecal contamination during milking (Zastempowska et al., 2016). The presence of pathogens in milk can vary considerably and is influenced by a number of factors including farm size, animal number, hygiene, farm management practices, antibiotic use, geographic location, and season (Oliver et al., 2005).

Some of the most important foodborne pathogens in cow's milk and products derived from the milk of dairy cows are *Brucella* spp., *Coxiella burnetii*, STEC, *Salmonella* spp., *Listeria monocytogenes*, and *Staphylococcus aureus* (Gale et al., 2015; Oliver et al., 2005; van Asselt et al., 2013; Zastempowska et al., 2016). Other foodborne pathogens that have been found in raw milk and have strong links to illness in humans include *Yersinia* spp. and *Campylobacter* spp. (Oliver et al., 2005; Zastempowska et al., 2016). Additionally, *Bacillus cereus*, *Corynebacterium* spp., *Cryptosporidium parvum*, *Mycobacterium bovis*, *Streptococcus suis* subspecies *zooepidemicus*, tick‐borne encephalitis virus, and *Toxoplasma gondii* are also transmissible through cow's milk (Zastempowska et al., 2016).

Other pathogens with zoonotic potential that have been found in milk include *Prototheca* spp., which are often highly resistant to antimicrobial drugs (Zastempowska et al., 2016). There are also a number of other antimicrobial‐resistant bacteria that are of particular concern in raw milk: multidrug‐resistant *Salmonella enterica* serovar Typhimurium, resistant *Campylobacter* spp., resistant STEC, methicillin‐resistant *S. aureus* (MRSA), and ESBL‐producing or AmpC gene‐carrying bacteria (Zastempowska et al., 2016).

**Figure 3:**
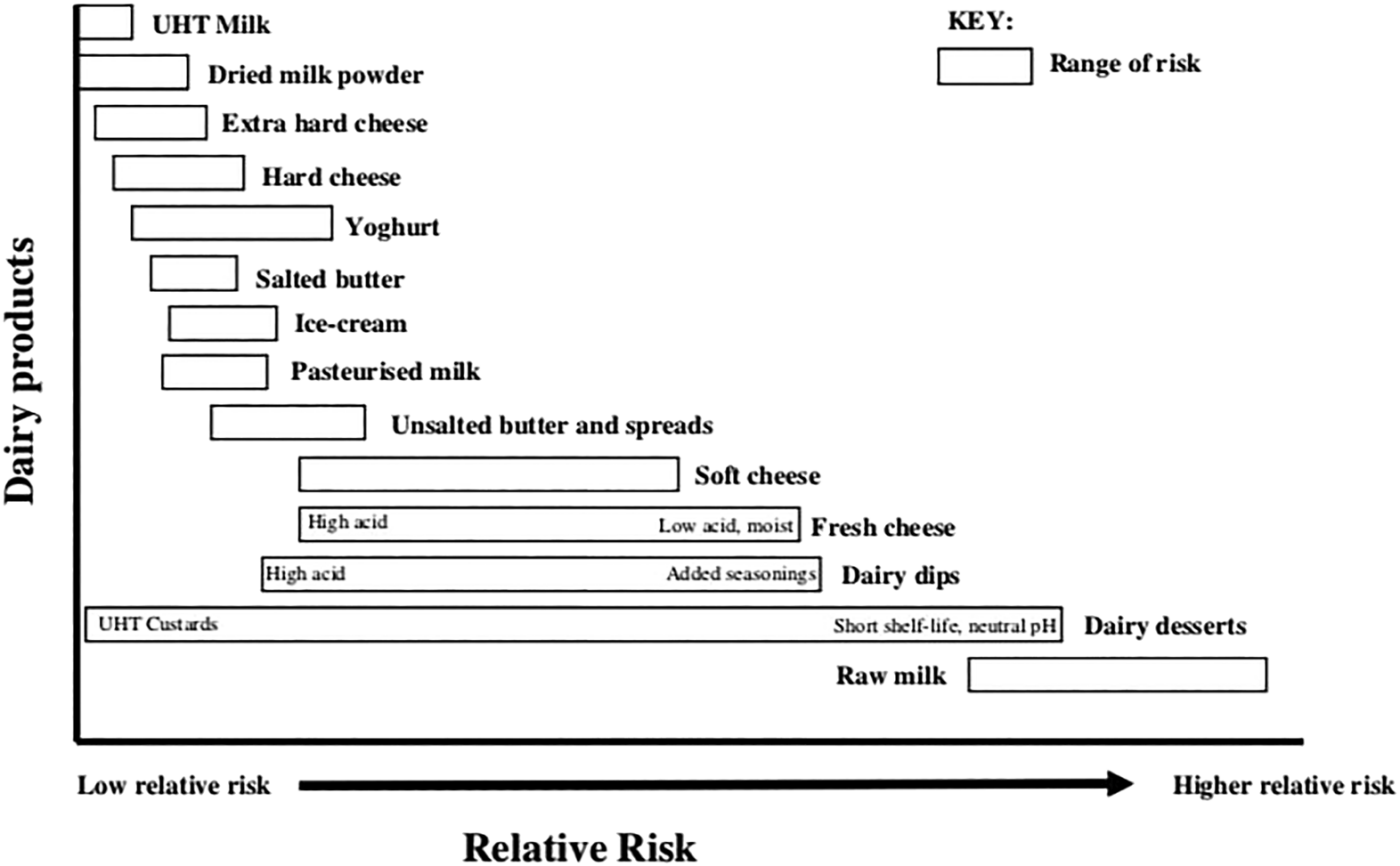
Relative risk from microbiological hazards in dairy products (FSANZ, 2006).

Different microbiological agents are of more concern for different processed dairy products (van Asselt et al., 2017). *E. coli* and *Campylobacter* spp. seem to be of particular concern in raw milk (van Asselt et al., 2017), while soft and semisoft cheeses were more frequently associated with *L. monocytogenes* and *S. aureus* (van Asselt et al., 2017). The contamination of powdered infant formula with *Cronobacter* spp. and *Salmonella* spp. is of particular concern due to the risk posed to infants (van Asselt et al., 2017). Furthermore, the risk from microbiological contamination of dairy products varies based on the intrinsic properties of each product (Figure 3) (FSANZ, 2006).

#### 6.1.1 Bacterial Toxins

Bacterial toxins in milk are also of concern, particularly those produced by *Bacillus* spp., *Clostridium* spp., and *Staphylococcus* spp. (Zastempowska et al., 2016). *Staphylococcus* spp. can produce staphylococcal enterotoxins (SE), SE‐like toxins (SEl), and toxic shock syndrome toxins (Zastempowska et al., 2016). SEs can be produced in milk that is not cooled quickly or is not pasteurized appropriately (Zastempowska et al., 2016). Once formed, SEs are heat‐stable proteins that cannot be destroyed during pasteurization (Zastempowska et al., 2016). *Bacillus* spp. and *Clostridium* spp. are also able to survive pasteurization in spore form; both can cause spoilage or food poisoning (Zastempowska et al., 2016). *Bacillus cereus* enterotoxins that can cause food poisoning include hemolysin BL and nonhemolytic enterotoxin (Zastempowska et al., 2016).

*Clostridium perfringens enterotoxin* (CPE) can also cause food poisoning and *Clostridium botulinum* is the causative agent of botulism (Zastempowska et al., 2016). CPE is a potential bovine mastitis pathogen (Zastempowska et al., 2016) that has been detected in cow milk and is resistant to pasteurization (McAuley et al., 2014). CPE has been indicated as the causal agent in at least one outbreak of foodborne illness in Australia caused by contaminated cheese sauce (McAuley et al., 2014). There was also one confirmed and one suspected outbreak of CPE attributed to dairy products in the United States between 1998 and 2008 (Bennett et al., 2013). *Clostridium botulinum* is not considered to be a bovine mastitis pathogen, but multiplication in silage and in the gastrointestinal tract have been reported, indicating that contamination of the environment and raw milk is possible (Lindström et al., 2010). *C. botulinum* has been isolated from powdered infant formula and other dairy powders (Barash et al., 2010; Doyle et al., 2015), however contaminated powders have not conclusively been demonstrated to have caused outbreaks of human botulism (Doyle et al., 2015). Outbreaks of human botulism have been associated with other dairy products such as cheese, milk, and yogurt, although such outbreaks are rare (Doyle et al., 2015; Lindström et al., 2010). Shiga toxins produced by *E. coli* strains can cause severe tissue damage in humans, particularly in children, and have been associated with diarrhea, hemorrhagic colitis, thrombotic thrombocytopenia purpurea, and hemolytic uremic syndrome (Zastempowska et al., 2016). Cattle are considered the primary reservoir for STEC globally (Zastempowska et al., 2016).

#### 6.1.2 Burden of Bacterial Foodborne Illness Attributable to Dairy

A World Health Organization structured expert elicitation study calculated global estimates of the proportion of foodborne illnesses attributable to specific foods (Hoffmann et al., 2017). Contaminated dairy products were estimated to cause approximately 68–91% of foodborne brucellosis cases; 15% of foodborne STEC cases; 4–15% of foodborne campylobacteriosis cases; 2–6% of foodborne nontyphoidal salmonellosis cases; and 2–8% of foodborne cryptosporidiosis cases (Hoffmann et al., 2017). However, only 8–16% of global cryptosporidiosis cases were estimated to be foodborne (Hald et al., 2016; Hoffmann et al., 2017). By contrast, approximately 44–75% of total brucellosis cases, 40–60% of total STEC cases, 51–76% of total campylobacteriosis cases, and 46–73% of total nontyphoidal salmonellosis cases were estimated to be foodborne across all regions (Hald et al., 2016; Hoffmann et al., 2017). These estimates have broad uncertainty bounds due to a lack of data in many countries and the results are difficult to compare to other studies, especially as prior studies have used different points of attribution (Hoffmann et al., 2017). However, several smaller scale studies conducted in North America and Europe have provided fairly similar estimates for percentages of foodborne illnesses attributable to dairy products (Davidson et al., 2011; Havelaar et al., 2008; Hoffmann et al., 2007; Hoffmann et al., 2017; Painter et al., 2013).

One such study, conducted in the United States, estimated annual foodborne illnesses, hospitalizations, and deaths attributable to different food commodities for the period from 1998 through 2008 (Painter et al., 2013). In total, the study attributed 1.3 million (14%) illnesses, of the 9.6 million estimated annual illnesses, to dairy products each year (Painter et al., 2013). Additionally, 16% of hospitalizations and 10% of deaths were attributed to dairy products (Painter et al., 2013). Only produce commodities, including fruits, vegetables, and nuts, caused more illnesses and only poultry caused more deaths (Painter et al., 2013). Dairy products resulted in the most hospitalizations of the commodities examined (Painter et al., 2013). Approximately 18% of bacterial illnesses were attributed to dairy, matching the percent attributed to poultry (Painter et al., 2013). Additionally, 12% of viral illnesses were attributed to dairy products, in large part due to the contamination of cheese with norovirus after pasteurization (Painter et al., 2013). However, the study may have overestimated the illnesses attributable to dairy, in part due to a large campylobacteriosis outbreak that was traced back to pasteurized milk (Painter et al., 2013). While these results are bounded by considerable uncertainty and are specific to the United States, it is possible that other high‐income nations with similar food systems may face a comparable burden of foodborne illnesses attributable to dairy.

### 6.2 Chemical Contaminants

The introduction of chemical contamination to dairy products frequently occurs during primary production, either through the consumption of contaminated feed by cattle, uptake of chemical compounds from the environment primarily through grazing on contaminated soils, through the administration of veterinary medicines, or due to fraud (van Asselt et al., 2017). Potential chemical hazards in milk and dairy products include antibiotics (e.g., beta lactams, sulphonamides, and tetracyclines), antihelmintics, and other veterinary drugs; cleaning agents used to disinfect or sanitize milk lines or equipment; and chemicals from environmental sources, including pesticides, heavy metals, dioxins, furans, polychlorinated biphenyls, and radionuclides (Smith, 2016; van Asselt et al., 2017). Mycotoxins, such as aflatoxin M1 (AFM1), that originate from contaminated feeds can also be transferred to milk (Smith, 2016). Furthermore, dairy products can be adulterated with chemical additives, such as melamine, in order to artificially simulate the appearance of increased nutrient content (Soon, 2016). The chemicals of most concern in dairy products include AFM1, dioxins and other environmental contaminants, and veterinary drug residues (van Asselt et al., 2017).

Mycotoxins are toxic chemical compounds produced by different genera of yeast or filamentous fungi (Becker‐Algeri et al., 2016; Smith, 2016; van Asselt et al., 2017). Mycotoxins are produced when cattle ingest contaminated feed and they are often excreted in milk (Becker‐Algeri et al., 2016; Cavirani, 2008; Smith, 2016; van Asselt et al., 2017). It has been estimated that at least 25% of all food crops are affected by mycotoxins (Cavirani, 2008). While the levels of mycotoxins excreted in milk are generally low, mycotoxins are heat resistant and can survive pasteurization (Becker‐Algeri et al., 2016). Mycotoxins that are of potential concern in milk and dairy products include aflatoxins, especially AFM1, fumonisins, zearalenone, ochratoxin A, tricothecenes, T‐2 toxin, and deoxynivalenol (Becker‐Algeri et al., 2016).

Aflatoxins are toxic, and potentially carcinogenic and immunosuppressive, fungal metabolites that are often produced by *Aspergillus flavus* (Becker‐Algeri et al., 2016; Cavirani, 2008). AFM1 is produced when cattle eat feed contaminated with aflatoxin B1 and has frequently been isolated from milk (Becker‐Algeri et al., 2016; Smith, 2016; van Asselt et al., 2017). The relationship between aflatoxin concentrations in food and acute illness (i.e., aflatoxicosis) has been established and has led to regulatory limits in many countries, but little is known about specific threshold levels associated with other adverse health effects (Strosnider et al., 2006). While a number of studies in certain countries (e.g., Brazil, China, Sudan, and Turkey) have reported aflatoxin levels in dairy products exceeding recommended or regulatory limits (Bahrami et al., 2015; Bilandžić et al., 2014; Elzupir & Elhussein, 2010; Iha et al., 2011; Raul et al., 2012; Temamogullari & Kanici, 2014; Xiong et al., 2018), the risk to human health is not clear. It has been estimated that aflatoxin exposure in nuts and maize may play a causative role in anywhere from 4.6–28.2% of global hepatocellular carcinoma, or liver cancer, cases (Liu & Wu, 2010). Therefore, it is possible that high aflatoxin levels in milk and dairy products may also play a causative role in a considerable portion of global liver cancer cases.

Uptake of a number of different chemical compounds by cattle during grazing is also a potential problem for dairy farms, especially in areas that are environmentally contaminated, as some chemicals can then be excreted in cow's milk (Smith, 2016; van Asselt et al., 2017). Organochlorines are highly toxic, persistent organic pollutants that can have adverse effects on human health and the environment (Avancini et al., 2013). These compounds are lipophilic and bioaccumulate in animal and human adipose tissue through the food web (Avancini et al., 2013; van Asselt et al., 2017). Organochlorine pollutants of concern include polycyclic aromatic hydrocarbons, dioxins, furans, polychlorinated biphenyls, perfluorinated compounds, and brominated flame retardants, which can cause serious health issues in humans (Smith, 2016; van Asselt et al., 2017). Pesticides are occasionally found in milk, especially organochlorine pesticides, such as dichlorodiphenyltrichloroethane, lindane, and hexachlorocyclohexanes (Smith, 2016; van Asselt et al., 2017). Other pesticides that are sometimes found in milk include organophosphorus pesticides, carbamates, and pyrethroids (Smith, 2016; van Asselt et al., 2017).

Other organic pollutants and heavy metals can contaminate the dairy farm environment as a result of fertilizer application, contaminated materials such as industrial waste or sewage sludge, or atmospheric deposition (van Asselt et al., 2017). Furthermore, nuclear accidents or explosions can lead to the deposition of radionuclides on pastures where cattle can then ingest them and they can be transferred to milk (van Asselt et al., 2017). For example, iodine‐131 was reported in milk following the Chernobyl accident and other radionuclides such as cesium‐137 have also been reported (van Asselt et al., 2017). Chronic exposure to radionuclides as a result of the ingestion of contaminated foods can increase cancer risk, although risk depends on the type of radionuclide, amount ingested, and duration of exposure (Olobatoke & Mathuthu, 2015). Additionally, different radionuclides may affect different organs or tissues (Olobatoke & Mathuthu, 2015). For example, chronic intakes of iodine‐131 can cause thyroid cancer (Murakami & Oki, 2012).

The true public health burden of foodborne illnesses caused by chemical agents that are attributable to dairy products is not clear; many of the attribution studies reviewed here only examined the burden of biological agents (Davidson et al., 2011; Havelaar et al., 2008; Hoffmann et al., 2007; Hoffmann et al., 2017). In the United States, a study estimated that approximately 3,773 foodborne illnesses, 23 hospitalizations, and 2 deaths were attributable to dairy products contaminated with chemical agents each year (Painter et al., 2013). However, the study results were limited by the lack of published estimates for a number of illnesses caused by chemical agents (Painter et al., 2013).

### 6.3 Foodborne Hazards Summary

Food safety hazards can affect the dairy supply chain at several different points, including through animal feed, through the farm environment, and through processing (Oliver et al., 2005; Smith, 2016; van Asselt et al., 2017; Zastempowska et al., 2016). Overall, the most commonly encountered hazards in milk and dairy products are microbiological and chemical hazards (Smith, 2016; van Asselt et al., 2017). In the near future, climate change and intensification of dairy farming may amplify food safety risks globally, particularly those associated with microbiological hazards and possibly certain chemical hazards. Increased temperatures and changes in humidity may influence the lifecycle of pathogens and mycotoxin production (Havelaar et al., 2010;Paterson & Lima, 2010 ; van Asselt et al., 2017). With increasing global demand for dairy products and the intensification of the global dairy sector, a corresponding increase in the demand for supplemental animal feed has been seen (OECD & FAO, 2016; van Asselt et al., 2017). This increased demand for animal feed coupled with climate change has raised concerns that mycotoxin occurrence in animal feed may increase in some regions (Paterson & Lima, 2010; van Asselt et al., 2017). These trends may have important implications for food safety in the future.

## 7 Diet‐Related Harms and Benefits

The impacts of agricultural production are driven in part by dietary choices (Tilman & Clark, 2014). Despite the intensification and expansion of agriculture, almost one billion people have inadequate diets and lack secure food supplies (Godfray et al., 2010; Tilman & Clark, 2014). At the same time, rising incomes and urbanization are driving a global nutrition transition toward diets higher in processed food, refined sugars and fats, oils, and animal products, which has contributed to an obesity epidemic, with more than two billion people becoming overweight or obese (Popkin et al., 2012; Tilman & Clark, 2014). This section aims to explore potential nutritional health harms and benefits associated with the consumption of dairy products.

Milk is a complex colloidal matrix that contains milk fat, lactose, and a number of different components, and each component may have positive and/or negative health effects (Kliem & Givens, 2011; Rodrigues, 2013). Milk and dairy products play an important role in human nutrition and are a key source of protein, vitamins, and minerals (Hess et al., 2016;Kliem & Givens, 2011 ; Pereira, 2014). However, concerns have been raised regarding the potential health hazards related to the fat content of milk (Haug et al., 2007; Melnik, 2009; Pereira, 2014; Rodrigues, 2013) and the naturally occurring hormones found in milk (Malekinejad & Rezabakhsh, 2015; Melnik, 2009). Generally, the epidemiological evidence indicates that the moderate consumption of dairy products does not increase the risk for most chronic conditions and may actually confer protection against cardiovascular disease, stroke, diabetes, dementia, certain cancers, and mortality (Dehghan et al., 2018; Haug et al., 2007;Hess et al., 2016 ; Kliem & Givens, 2011 ; Pereira, 2014). The definition of moderate consumption varied between studies, but most of the studies reviewed here loosely defined moderate consumption as between one to three servings of dairy per day (e.g., Haug 2007, Elwood 2010, Hess 2016), with a serving commonly defined as approximately one cup (244 g) of milk or yogurt, or one slice of cheese (42.5 g). Generally, the measurements used to indicate moderate consumption fell within the recommended dietary guidelines issued by various countries; many nations recommend consuming at least one serving of dairy per day (Hess et al., 2016).

### 7.1 Composition of Milk

Dairy products are estimated to contribute approximately 20% of total fat consumed in Western diets and more than 50% of energy from milk is derived from fat (Kliem & Givens, 2011). Around 70% of milk fat is composed of saturated fatty acids (Kliem & Givens, 2011). Milk also contains high‐quality protein (Hess et al., 2016; Kliem & Givens, 2011), although the fat and protein content of milk varies by cow breed and nutrition (Kliem & Givens, 2011). Additionally, milk contains vitamins, minerals, and other components that may benefit health including bioactive peptides, milk fat globule membrane, prebiotics, and probiotics (Hess et al., 2016). While whole milk naturally contains a number of vitamins and minerals, milk can also be fortified with additional vitamins and minerals during processing. For example, in some countries milk is fortified with vitamins A and D (Pereira, 2014). Selenium and iodine can also be added to cattle feed to boost levels in milk (Haug et al., 2007). A number of articles examined the nutrient profile of dairy products, and Table 2 provides a list of important vitamins and minerals and the average concentrations in whole cow's milk.

**Table 2:** Nutrient Content of Cow's Milk

Nutrient	Content per cup (244 g) of whole milk fortified with vitamin D^a^
Vitamin A	112 μg
Vitamin B1 (thiamin)	0.112 mg
Vitamin B2 (riboflavin)	0.412 mg
Vitamin B3 (niacin)	0.217 mg
Vitamin B6 (piridoxin)	0.088 mg
Vitamin B12 (cobalamin)	1.10 μg
Vitamin D	124 μg
Vitamin E	0.17 mg
Folate	12 μg
Calcium	276 mg
Iodine	38 μg^b^
Magnesium	24 mg
Phosphorus	205 mg
Potassium	322 mg
Selenium	9 μg
Zinc	0.90 mg

*Note*. This table was created using estimates from Haug et al. (2007), Kliem and Givens (2011), Pereira (2014), and Hess et al. (2016).

a According to the USDA National Nutrient Database for Standard Reference Release 28 (https://ndb.nal.usda.gov/ndb/foods/show/70).

b Levels of iodine in milk can vary significantly, value adapted from Haug et al. (2007).

### 7.2 Cow's Milk Consumption in Infants and Children

Consumption of cow's milk in infants and toddlers is a risk factor for the development of iron deficiency anemia (IDA) (Griebler et al., 2016; Parkin et al., 2016). A systematic review and meta‐analyses reported that there is a more than threefold increased risk for IDA for children 8–18 months old who were fed cow's milk as compared to those that were fed fortified formula for 6–12 months from either birth or 6 months of age (Griebler et al., 2016). IDA occurs in approximately 1–2% of infants in high‐income nations and typically peaks at 1–3 years of age (Parkin et al., 2016). Children with severe IDA experience substantial morbidity including developmental delays, heart failure, and stroke (Parkin et al., 2016). Children with severe IDA also have increased health care utilization, including hospitalization and blood transfusion (Parkin et al., 2016). Generally, infants introduced to cow's milk before the age of one and children ages 1–5 years who drink more than 700 ml of cow's milk each day are at increased risk of iron deficiency (MFMER, 2017). It is recommended that the introduction of cow's milk be delayed until after twelve months of age and after that cow's milk consumption should be limited to 500 ml/day (Parkin et al., 2016).

### 7.3 Cow's Milk Consumption in Adults

For adults, there is concern that the saturated fatty acid content of milk and dairy products may contribute to cardiovascular disease, weight gain, and obesity (Haug et al., 2007; Pereira, 2014; Rodrigues, 2013). Additionally, some researchers have hypothesized that the hormone content of milk may play a role in the development of certain forms of cancer (Malekinejad & Rezabakhsh, 2015). However, there are a number of different components in milk that all have different effects on health and disease etiology (Haug et al., 2007; Pereira, 2014) and a number of components of milk, such as oleic acid, conjugated linoleic acid, omega‐3 fatty acids, proteins, vitamins, minerals, and bioactive peptides may provide health benefits (Haug et al., 2007; Rodrigues, 2013). For example, conjugated linoleic acid may prevent cardiovascular disease and benefit immunity (Pereira, 2014); and lactoferrin may inhibit the growth of tumors and reduce susceptibility to cancer (Rodrigues et al., 2008; Zhang et al., 2014). Other components of milk and dairy products may have positive and/or negative effects on the digestive, cardiovascular, and nervous systems (Haug et al., 2007; Hess et al., 2016; Kliem & Givens, 2011; Rodrigues, 2013). The links between a number of different diet‐related chronic diseases and the consumption of dairy products are reviewed below.

#### 7.3.1 Cardiovascular Disease

Diets high in saturated fatty acids are generally thought to contribute to cardiovascular disease and more than half of the fatty acids in milk are saturated (Haug et al., 2007). Some of the saturated fatty acids found in cow's milk, including lauric, myristic, and palmitic acids, have been associated with increases in blood cholesterol levels (Haug et al., 2007). However, diets containing low‐fat dairy products have been consistently associated with improved serum cholesterol levels (Haug et al., 2007). Additionally, a number of epidemiological and intervention studies have reported an inverse relationship between milk consumption and hypertension (Haug et al., 2007; Hess et al., 2016), which may be due to the antihypertensive effects of certain milk peptides (Haug et al., 2007). Studies have not generally shown an increased risk for cardiovascular disease among people with higher dairy fat intake (Haug et al., 2007; Kliem & Givens, 2011; Smedman et al., 1999; Warensjo et al., 2004), and a number of epidemiological cohort studies suggest that the moderate consumption of milk and dairy products may protect against cardiovascular disease (Dehghan et al., 2018; Elwood et al., 2005; Sauvaget et al., 2003; Sun et al., 2014).

The Prospective Urban Rural Epidemiology study was the first large, multinational cohort study to examine the associations between dairy consumption and mortality and cardiovascular disease (Dehghan et al., 2018). The Prospective Urban Rural Epidemiology study included 136,384 diverse individuals, aged 35–70 years, from 21 countries on five continents (Dehghan et al., 2018). Specifically, participants were from Argentina, Bangladesh, Brazil, Canada, Chile, China, Colombia, India, Iran, Malaysia, occupied Palestinian territory, Pakistan, Philippines, Poland, South Africa, Saudi Arabia, Sweden, Tanzania, Turkey, United Arab Emirates, and Zimbabwe (Dehghan et al., 2018). Information on food intake was collected using country‐ or region‐specific validated food frequency questionnaires (Dehghan et al., 2018). The study found that dairy consumption was significantly associated with lower risk of mortality and major cardiovascular disease events (Dehghan et al., 2018). The risk of stroke was also significantly lower with higher dairy consumption (i.e., more than two servings per day; Dehghan et al., 2018). However, myocardial infarction was not significantly associated with dairy intake (Dehghan et al., 2018). The study's findings supported the hypothesis that the consumption of dairy products may be beneficial for mortality and cardiovascular disease, especially in low‐ and middle‐income nations where dairy intake tends to be substantially lower than in Europe and North America (Dehghan et al., 2018).

Recent meta‐analyses (Soedamah‐Muthu et al., 2011) and reviews (Hess et al., 2016; Kliem & Givens, 2011; Pereira, 2014) have also indicated that moderate dairy consumption does not contribute to the development of cardiovascular disease. Overall, evidence indicates that moderate milk and dairy product consumption has a protective influence on cardiovascular disease, although consumption of low or reduced fat dairy products over full fat products may be recommended for high risk populations (Pereira, 2014).

#### 7.3.2 Type 2 Diabetes, Metabolic Syndrome, and Obesity

Moderate milk and dairy consumption also seem to be associated with a reduced risk for type 2 diabetes and associated weight problems, including obesity and metabolic issues (Haug et al., 2007;Hess et al., 2016 ; Pereira, 2014). Metabolic syndrome has a number of different risk factors including abdominal obesity, high triglyceride levels, high blood pressure, insulin resistance, inflammation, and prothrombotic state, all of which increase the risk for a number of other conditions such as type 2 diabetes and cardiovascular disease (Hess et al., 2016). Recent reviews of the most common risk factors, including central obesity, high blood pressure, and hyperglycemia, found that moderate dairy consumption is associated with a lower risk of central obesity (Crichton & Alkerwi, 2014; Hess et al., 2016) and may also lower blood pressure (Fumeron et al., 2011; Hess et al., 2016). Furthermore, the risk of type 2 diabetes may be lower for individuals with a higher dairy intake than for those with low dairy intake (Elwood et al., 2010; Kliem & Givens, 2011). Evidence for the effect of dairy intake on blood glucose levels is inconclusive (Hess et al., 2016). However, one study posits that theoretically milk consumption may induce postprandial hyperinsulinemia and may lead to a permanent increase in insulin‐like growth factor 1 serum levels (Melnik, 2009). This effect may be a cause for concern because insulin and insulin‐like growth factor 1 signaling may play a role in the regulation of fetal growth, T‐cell maturation in the thymus, pathogenesis of acne, atherosclerosis, diabetes mellitus, obesity, cancer, and neurodegenerative diseases (Melnik, 2009).

#### 7.3.3 Musculoskeletal Health

Moderate milk consumption may promote bone density and protect against osteoporosis (Hess et al., 2016; Pereira, 2014), since milk and dairy products have high levels of bioavailable calcium compared with other foods (Hess et al., 2016). Regular dairy intake is often recommended for the prevention of osteoporosis, but while recent evidence points to a link between dairy consumption and bone health, the dose‐response relationship remains unclear (Hess et al., 2016). It is possible that dairy consumption may benefit bone mass density in populations that have habitually low calcium intake, but it is difficult for researchers to separate the effect of calcium from the impact of dairy consumption (Hess et al., 2016). Age‐related loss of muscle bulk and strength (i.e., sarcopenia) is often linked to osteoporosis and there is some evidence that the consumption of additional dairy protein may contribute to the preservation of lean muscle mass in older adults (Hess et al., 2016). This effect may be due to the fact that milk and dairy products contain all nine essential amino acids and are bioavailable and digestible, despite containing fewer grams of protein than meat or beans (Hess et al., 2016). However, the consumption of additional dairy protein may only support the prevention of sarcopenia rather than amelioration of the condition (Hess et al., 2016).

#### 7.3.4 Cancer

The effect of milk and dairy consumption on different forms of cancer is often unclear due to the complex and multifactorial etiology of cancer and to the diverse effects of different components of milk (Pereira, 2014). Naturally occurring hormones and growth factors in milk may play a role in the etiology of certain forms of cancer (Malekinejad & Rezabakhsh, 2015; Pereira, 2014). A number of different food products contain naturally occurring hormones, but previous studies have estimated that as much as 60–80% of estrogens in Western diets come from milk and dairy products (Malekinejad & Rezabakhsh, 2015). It is possible that naturally occurring hormones and growth factors from milk intake may induce physiological responses, including stimulating the proliferation of epidermal, epithelial, and embryonic cells; inhibition of gastric acid secretion; promotion of wound healing; and bone resorption (Rodrigues, 2013). Hormones in milk and dairy products may act as endocrine disrupters and could potentially promote the development of prostate, breast, and endometrial tumors (Malekinejad & Rezabakhsh, 2015). However, more research is needed to determine the role of steroid hormones, especially estrogens, in the etiology of specific cancers (Malekinejad & Rezabakhsh, 2015).

Overall, there is mixed evidence regarding the relationship between dairy consumption and different forms of cancer. The varied actions of the different components of milk make it difficult to clarify the role of milk consumption in the development of certain cancers (Pereira, 2014). Current evidence indicates that the consumption of milk and dairy products may reduce the risk of colorectal cancer (Cho et al., 2004; Kliem & Givens, 2011; Larsson et al., 2006; Murphy et al., 2013; Pereira, 2014; Thorning et al., 2016) and bladder cancer (Kliem & Givens, 2011; Pereira, 2014), while there is conflicting evidence for the role of dairy consumption in the etiology of breast (Bessaoud et al., 2008; Pereira, 2014; Thorning et al., 2016; Zhang et al., 2011) and prostate cancer (Huncharek et al., 2008; Kliem & Givens, 2011; Pereira, 2014; Thorning et al., 2016). Chemical contaminants, such as pesticides, may play a role in the observed heterogeneity of study findings (Davoodi et al., 2013; Outwater et al., 1997).

#### 7.3.5 Dementia and Cognition

Dairy product consumption has been associated with improved cognition (Crichton et al., 2010; Crichton et al., 2011; Hess et al., 2016; Park & Fulgoni, 2013; Yamada et al., 2003), although the association may depend on the fat content and type of dairy product (Crichton et al., 2010; Park & Fulgoni, 2013). The mechanism by which dairy provides protection against dementia is unknown (Crichton et al., 2011; Kliem & Givens, 2011; Yamada et al., 2003), although it is possible that the link is due in part to the blood pressure regulating properties of milk (Crichton et al., 2011; Kliem & Givens, 2011). Additionally, some studies have attributed the link to the angiotensin converting enzyme inhibitors in bioactive peptides (Hess et al., 2016; Kris‐Etherton et al., 2009), but more research is needed to determine how dairy consumption influences cognitive health.

#### 7.3.6 Gastrointestinal Health

The moderate consumption of milk and other dairy products may also support digestive system health (Hess et al., 2016). Dairy products often contain prebiotics, which are food components that are primarily fermented in the intestine and promote the growth of beneficial microorganisms such as bifidobacteria and lactobacilli, and probiotics, which are live organisms that confer health benefits (Hess et al., 2016). For example, some lactic acid bacteria have been associated with beneficial health effects and may aid in digestion and reduce allergy risk (Zastempowska et al., 2016). Both prebiotics and probiotics contribute to improved digestive health by promoting the diversity and modulation of intestinal bacteria, which can contribute to protection from diarrheal diseases, inhibition of pathogenic infections, and constipation relief (Hess et al., 2016).

#### 7.3.7 Dietary Intolerance and Metabolic Disorders

Lactose intolerance symptoms (Haug et al., 2007; Pereira, 2014), galactosemia (Haug et al., 2007), and cow's milk protein allergy, which usually manifests in children and normally remits in adulthood (Haug et al., 2007; Pereira, 2014), are potential adverse health outcomes associated with dairy intake. Lactose intolerant individuals do not produce enough beta‐galactosidase (i.e., lactase), the enzyme required to hydrolyze lactose into glucose and galactose so that they can be absorbed into the small intestine (Hess et al., 2016). It is estimated that lactose intolerance occurs in more than 70% of the global population, although it is lower in some regions (Haug et al., 2007; Pereira, 2014). In the United States, it is estimated that approximately 25% of the population experiences some level of lactose intolerance (Haug et al., 2007). Lactose intolerance can result in abdominal cramps, bloating, flatulence, diarrhea, nausea, and vomiting (Pereira, 2014), although symptoms are usually not so severe as to require the exclusion of all milk and dairy from the diet for most people (Haug et al., 2007).

Another potential health effect linked to milk and dairy consumption is galactosemia, although this disorder is rare (Haug et al., 2007). Galactosemia is a metabolic disorder in which a person cannot properly metabolize the sugar galactose and the digestion of lactose in the intestines increases galactose concentrations (Haug et al., 2007). Galactosemia may lead to early onset cataracts and may also have negative effects on ovarian function and fertility (Haug et al., 2007).

### 7.4 Diet‐Related Health Harms and Benefits Summary

Dietary choices both influence and are influenced by agricultural production and the social environment (Tilman & Clark, 2014). The world is facing the double burden of malnutrition, which is characterized by the coexistence of under nutrition and over nutrition (Tilman & Clark, 2014). The role of dairy consumption in this double burden is not entirely clear. Dairy products play a crucial role in human nutrition and are an important source of protein, vitamins and minerals (Hess et al., 2016; Kliem & Givens, 2011; Pereira, 2014), but concerns have been raised regarding the potential health harms associated with the consumption of milk and dairy products (Haug et al., 2007; Malekinejad & Rezabakhsh, 2015; Melnik, 2009; Pereira, 2014; Rodrigues, 2013). Overall, the majority of recent studies suggest that the moderate consumption of dairy products does not increase the risk for most chronic conditions and may actually provide protection against cardiovascular disease, stroke, certain forms of cancer, and mortality (Dehghan et al., 2018; Haug et al., 2007; Hess et al., 2016; Kliem & Givens, 2011; Pereira, 2014). However, there is substantial heterogeneity in the literature and even the strongest associations between dairy consumption and beneficial health effects were countered by studies with contrasting results (Hess et al., 2016).

## 8 Economic, Social, and Cultural Impacts

Livestock keeping and production can indirectly influence public health through social, economic, and cultural impacts. The context of a person's life determines their health and wellbeing (Marmot et al., 2008; WHO, 2018b). Income and economic status, social status, social support networks, cultural customs and traditions, and gender, are all important determinants of health and wellbeing (Marmot et al., 2008; WHO, 2018b). Livestock keeping and production can influence these determinants of health in a number of different ways, although the impacts can vary considerably between lower‐income and higher‐income regions. Livestock production, including dairy farming, in lower‐income regions, especially in rural areas, can provide a number of beneficial services and outputs (Bettencourt et al., 2015; FAO et al., 2006; LID, 1999; Owen et al., 2005; Randolph et al., 2007). Livestock keeping has also become a common poverty reduction tool (Owen et al., 2005; Perry & Grace, 2009).

Livestock production systems managed by the poor in lower‐income nations tend to have substantially lower productivity per animal or per land unit than livestock production systems in industrialized nations (Randolph et al., 2007). Smallholder management systems are usually low or no input systems in which animals forage for themselves (Randolph et al., 2007). These systems also tend to include a mix of different livestock species to provide protection against risk (Randolph et al., 2007). By contrast, livestock production systems in higher‐income regions are typically characterized by a drive for higher productivity and efficiency, which often leads to the consolidation and intensification of operations (FAO et al., 2006; Randolph et al., 2007; Thornton, 2010).

### 8.1 Lower‐Income Nations and Regions

Livestock, including dairy cattle, are a main component of wealth in lower‐income nations and there is a link between livestock ownership, household economic status, and social welfare (LID, 1999; Thumbi et al., 2015). Livestock keeping does not require a formal education, nor does it always require large amounts of capital or land ownership (FAO et al., 2006). Therefore, it is often the only economic activity accessible to poor people in low‐income regions (FAO et al., 2006). An estimated two thirds of resource‐poor rural households keep some type of livestock (LID, 1999). The livestock systems managed by resource‐poor households typically reflect a number of resource constraints, such as limited financial resources, access to information, and access to services, as well as the lack of land ownership (Randolph et al., 2007).

Studies have specifically identified dairy farming as a critical source of livelihoods and nutritional security in a number of different lower‐income communities, nations, and regions, such as parts of Sub‐Saharan Africa (Chagunda et al., 2016; Kidoido & Korir, 2015; Rao et al., 2016; Ulicky et al., 2013); Bangladesh (Rahman, 2007); the Gaza Strip (Rossignoli et al., 2015); Inner Mongolia, China (Kiminami, 2009); Nepal (Singh & Maharjan, 2005); and India (Singh & Datta, 2013). Furthermore, an FAO review found that across household‐level randomized control trials and observational studies with a comparison group, dairy cow ownership and/or the improvement of dairy cow production had a consistent positive impact on indicators of household welfare around the world (FAO et al., 2018). The results were consistent across all study types, countries, and indicators and overall, the review provided strong evidence that dairy production makes a significant contribution to poverty reduction at both the household and community levels (FAO et al., 2018).

Livestock keeping is a particularly important income‐generating activity for women in low‐income regions and livestock represent one of the most widely held assets for women around the world (LID, 1999). For example, it was estimated that 75 million women are engaged in dairy farming in India, while by contrast only 15 million men are engaged in dairy farming (Ramkumar et al., 2004). Female dairy farmers in Pondicherry, India reported in a survey that owning dairy cattle facilitated the borrowing of money, offered security, conferred employment and provided food, milk, and dung (Ramkumar et al., 2004). Furthermore, the women who participated in the survey considered cattle to be integral assets, second in value to the huts they owned and their most important resource, aside from family labor (Ramkumar et al., 2004). Globally, women typically own and tend smaller livestock species, but larger draft animals can also help to reduce labor requirements for women, even if they are not directly owned (Owen et al., 2005).

Livestock are often kept by resource‐poor households for a variety of reasons, including for food production, income generation, use as financial instruments, provision of manure for fertilizer or fuel, production of power (e.g., use for transport or for plowing), and to enhance and reinforce social status and ties (Bettencourt et al., 2015; FAO, 2016; LID, 1999; Owen et al., 2005; Randolph et al., 2007). Specifically, livestock can provide a regular supply of food, which can either be consumed by the household or sold to generate regular income or occasional cash (Bettencourt et al., 2015; FAO, 2016; Owen et al., 2005; Randolph et al., 2007).

Improved food security is a particularly important benefit that can arise from livestock keeping. Dairy cattle can be a source of milk and meat. However, the links between livestock keeping and human health and nutritional status are numerous and complex (Randolph et al., 2007; Thumbi et al., 2015). Generally, keeping healthy livestock can help to reduce the burden of disease, improve household nutritional status, and can also lead to increased household income, wealth, access to education, and access to health care (Thumbi et al., 2015). However, livestock production can also harm human health and nutritional status through indirect social and economic pathways (Randolph et al., 2007). For example, the allocation of household resources such as land and labor to livestock production can, under certain circumstances, decrease production and in turn decrease both the consumption and sale of food (Randolph et al., 2007). Household labor that is allocated to livestock production can also increase overall household labor demands, particularly for women (Randolph et al., 2007). This can lead to a reduction in the time and quality of care and feeding for young children, impacting their nutritional status (Randolph et al., 2007).

In lower‐income regions, livestock are important sources of income generation and can be used as financial instruments (Bettencourt et al., 2015; FAO, 2016; LID, 1999; Owen et al., 2005; Randolph et al., 2007). Specifically, livestock can provide a way for the poor to store savings or accumulated capital when they lack access to banks and standard financial markets or instruments (Bettencourt et al., 2015; Owen et al., 2005; Randolph et al., 2007). Livestock can also be sold and transformed into cash, providing instruments of liquidity and consumption smoothing (i.e., creating stable and predictable spending patterns across periods of varied income generation) (Bettencourt et al., 2015; Owen et al., 2005; Randolph et al., 2007). Additionally, livestock can serve as a form of insurance, providing a household with assets that can be sold in an emergency (Bettencourt et al., 2015; Owen et al., 2005; Randolph et al., 2007).

Livestock manure can be used to improve or maintain soil fertility and contribute to greater crop production for food and additional income generation (Bettencourt et al., 2015; FAO, 2016; Owen et al., 2005; Randolph et al., 2007). Animal manure supplies around 15% of nutrients applied as crop fertilizer globally (FAO, 2016). Additionally, in some areas manure is used as solid fuel or for the generation of biogas (FAO, 2016; Randolph et al., 2007). Dung can also be used as a building material and is often a marketable commodity (Bettencourt et al., 2015; FAO, 2016; Owen et al., 2005; Randolph et al., 2007). Livestock can also provide power for transportation or be used in place of farm equipment for crop production (Bettencourt et al., 2015; FAO, 2016; Owen et al., 2005; Randolph et al., 2007). Approximately two billion people in lower‐income nations rely on livestock for draft power and transportation (FAO, 2016).

#### 8.1.1 Poverty Reduction in Lower‐Income Nations and Regions

Dairy farming, and livestock keeping in general, is considered an important tool for poverty reduction in lower‐income areas (FAO et al., 2018). Poverty is defined as not only the lack of money or materials, but as a multidimensional social phenomenon that can range from material deprivation to the psychological experience of multiple deprivations (Owen et al., 2005). For example, poverty has consistently been tied to undernutrition, lack of access to safe water, and lack of access to health services (Corvalan et al., 2005). There is a general consensus that economic growth is crucial for poverty reduction, but economic growth alone is typically insufficient to reduce poverty on a broader scale (Perry & Grace, 2009). Poverty reduction also requires favorable conditions for entrepreneurial investment, lack of corruption, and improved governance in public and private sectors, as well as transparency and accountability (Perry & Grace, 2009). Additionally, in order for the benefits of economic growth to be realized, investments in poverty reduction must be coupled with the development of policies that support the delivery of education and health services, and improve social infrastructure (Perry & Grace, 2009). Absolute levels of poverty, as well as economic disparities within a population, are important components of health and wellbeing (Marmot et al., 2008; Perry & Grace, 2009).

Livestock, including dairy cattle, are important to both the urban and rural poor (Owen et al., 2005; Perry & Grace, 2009) and can contribute to poverty reduction in a number of different ways (FAO et al., 2018; Perry & Grace, 2009). Generally, livestock can serve as assets that help to meet livelihood needs or as safety nets, especially for vulnerable populations (e.g., the poorest, women, and those who are immunocompromised), and provide a pathway out of poverty (Perry & Grace, 2009). Livestock can also contribute to the livelihoods of those who do not keep livestock (Perry & Grace, 2009). For example, there are a number of people involved in the value addition of livestock products around the world (Perry & Grace, 2009). Case studies suggest that animal‐based foods are among the most commonly sold street foods and are often sourced from animals kept in cities (FAO, 2005; Perry & Grace, 2009). Moreover, the street food sector is often an important informal sector employer (FAO, 2005; Perry & Grace, 2009). For example, the street food sector is the largest informal sector employer in South Africa and may be a major contributor to the South African economy (Perry & Grace, 2009; Von Holy & Makhoane, 2006). There are also a large number of poor people who consume livestock products, even if they are not involved in livestock keeping (Perry & Grace, 2009).

**Figure 4:**
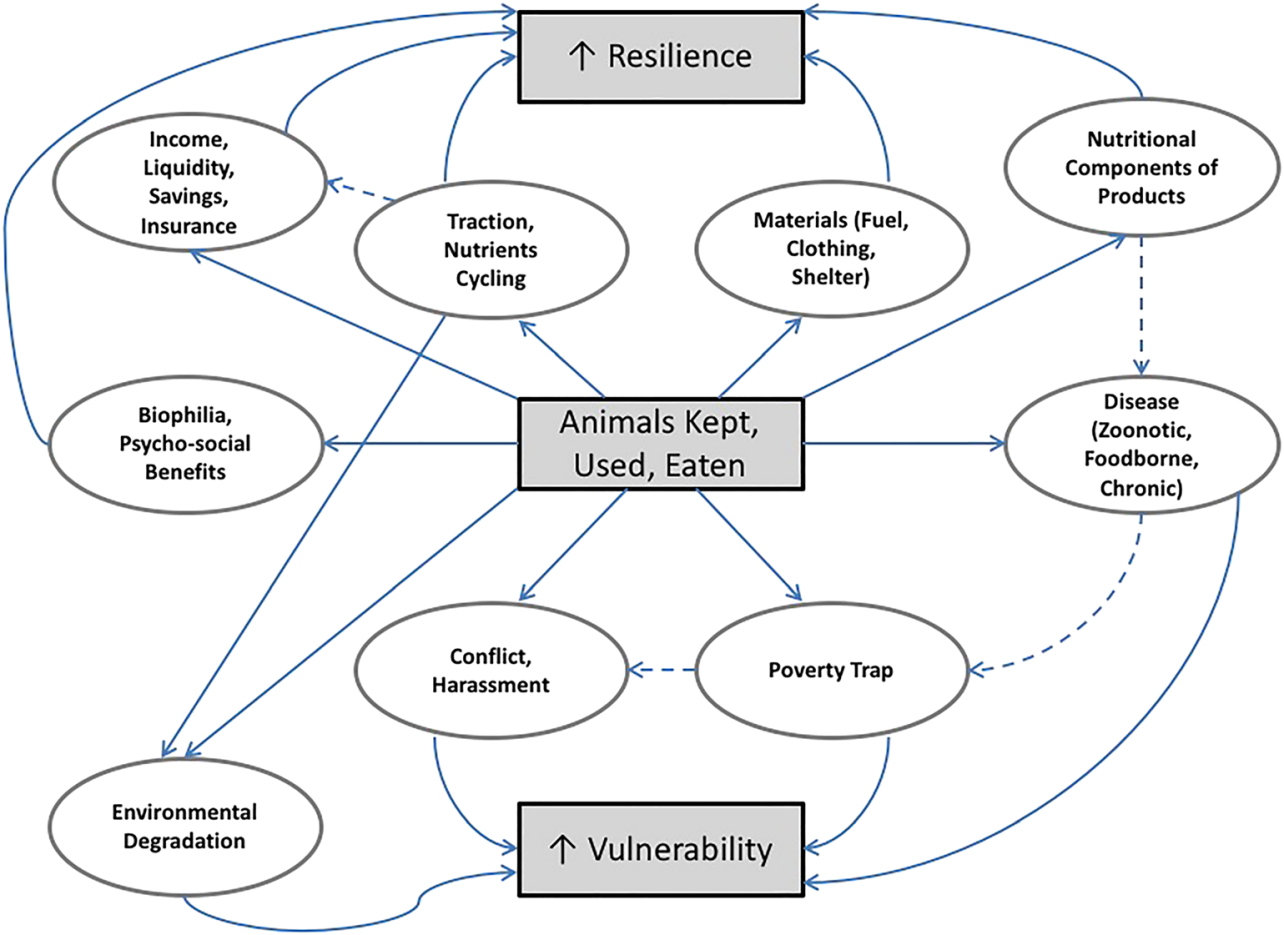
Positive and negative impacts of livestock on livelihoods. Solid arrows indicate a direct link; dashed arrows indicate an indirect link. Adapted from Perry and Grace (2009).

Overall, the specific context in which livestock can reduce household poverty is complex and there are numerous linkages between livestock production in low‐income settings and human health and wellbeing (Figure 4) (Perry & Grace, 2009). Additionally, while there are a number of ways in which keeping livestock can increase resilience and improve livelihoods, keeping livestock can also increase vulnerability and undermine livelihoods and wellbeing under certain circumstances (Perry & Grace, 2009).

#### 8.1.2 Other Social and Cultural Links and Impacts

Livestock keeping and production may also affect human health and wellbeing through other social and cultural impacts. In many societies, livestock are considered an indicator of social importance within a community (Bettencourt et al., 2015; FAO, 2016; Randolph et al., 2007). Animals that are given as gifts often play an important role in social relations and events, such as rites of passage, weddings, and funerals (Bettencourt et al., 2015; FAO, 2016). Dairy cattle and other livestock are also used as dowry or bride price in some communities (Bettencourt et al., 2015; FAO, 2016; FAO et al., 2006). Additionally, animal sacrifice and the consumption of animal products frequently play a part in religious ceremonies around the world (Bettencourt et al., 2015; FAO, 2016).

Certain livestock species and breeds are sometimes closely tied to the cultural identity of a people, as evidenced by their prominent roles in creation stories and oral histories (FAO, 2016). Many livestock species and breeds are also associated with unique indigenous knowledge systems and management practices (FAO, 2016). There are 16 United Nations Educational, Scientific and Cultural Organization (UNESCO) World Heritage Sites and three Globally Important Agricultural Heritage Systems that are directly linked to pastoral systems (FAO, 2016).

Farmland can provide open space for recreation as well as valuable habitats for certain wildlife species. It may also add esthetic value to some landscapes (Pretty et al., 2000; Tegtmeier & Duffy, 2004). Artists, writers, and philosophers throughout history have drawn inspiration from pastoral landscapes and livestock often play a crucial role in shaping the landscape (FAO, 2016). Humans may have an innate tendency to seek interactions with nature and animals, also referred to as biophilia (Wilson, 1984). Traditions surrounding livestock keeping can also attract tourists and may provide the basis for eco‐ or agrotourism activities (FAO, 2016).

### 8.2 Higher‐Income Nations and Regions

Livestock production systems in higher‐income nations differ considerably from the smallholder systems typical in lower‐income nations. In higher‐income nations, a smaller proportion of the population is engaged in livestock keeping and production as long‐term structural changes to the sector lead to consolidation (FAO et al., 2006; Randolph et al., 2007; Thornton, 2010). The shift toward the intensification of livestock production systems is driven by economies of scale (FAO et al., 2006). The shift is typically accompanied by an increase in the size of operations, which is usually achieved at the cost of forcing small and middle‐sized producers out of business (FAO et al., 2006). This trend has been witnessed globally and raises a number of social issues, including rural emigration and the concentration of wealth (FAO et al., 2006).

The shift toward larger operations specifically raises concerns about the vitality of rural communities. A review of community and socioeconomic issues associated with large agricultural operations, such as confined animal feeding operations, in the United States found that the economic concentration of agricultural operations tends to remove more money from rural communities than when there are more, smaller farming operations (Donham et al., 2007). The review specifically stated that communities with more small, owner‐operated farms had “richer civic and social fabric with more retail purchases made locally and with income more equitably distributed (Donham 2007, pg. 317).” There may also be economic effects at the household level. While farm profits generally increase with system intensity, in cases of extreme intensification, farm profit margins can decline as the cost of supplementary inputs rise (Doole & Romera, 2015; Macdonald et al., 2011; McCall & Clark, 1999).

Efforts to control land development trends, such as urbanization or the intensification of agricultural operations (e.g., the rise of confined animal feeding operations), can result in increases in local land use regulations (White, 2000; Wu, 2008). Land use regulation can take different forms and traditional command and control approaches include zoning, density regulation, and other direct land use controls (Wu, 2008). However, the introduction of land use regulations may have unintended socioeconomic consequences (Wu, 2008). For example, studies conducted in the United States have shown there is a link between land use regulation and housing affordability (Cho et al., 2003; Glaeser & Ward, 2009; Wu, 2008). Specifically, land use regulation can lead to higher housing prices which can make housing less affordable for middle‐ and lower‐income households (Cho et al., 2003; Glaeser & Ward, 2009; Wu, 2008). While it is not clear whether land use regulations specifically targeted at agricultural land use would affect housing affordability or availability, this concern requires further research and consideration, especially during policy development.

While a number of the impacts associated with livestock production, including dairy farming, are markedly different in higher‐income regions, there are a number of effects that may be common across regions. Livestock keeping and production provide economic opportunities around the world, including employment and income generation (FAO, 2016), although there may be fewer opportunities in higher‐income regions due to intensification and consolidation within the sector (FAO et al., 2006; Randolph et al., 2007; Thornton, 2010). Additionally, as in lower‐income regions, livestock production in higher‐income regions may be associated with benefits related to leisure, recreation, tourism, education, and inspiration opportunities (FAO, 2016; Pretty et al., 2000; Tegtmeier & Duffy, 2004).

### 8.3 Economic, Social, and Cultural Impacts Summary

Livestock keeping and production can indirectly influence public health through social, economic, and cultural impacts. However, the effects can vary considerably from lower‐ to higher‐income nations, due in part to distinct livestock production system characteristics (FAO et al., 2006; Randolph et al., 2007; Thornton, 2010). In lower‐income nations, livestock, including dairy cattle, are a main component of wealth and can provide a variety of economic benefits (Bettencourt et al., 2015; LID, 1999; Owen et al., 2005; Randolph et al., 2007; Thumbi et al., 2015). Furthermore, livestock keeping has become a common poverty reduction tool (Owen et al., 2005; Perry & Grace, 2009). Livestock can also confer social status and strengthen social networks and cultural ties (Bettencourt et al., 2015; FAO, 2016; Randolph et al., 2007). By contrast, in higher‐income nations, fewer people tend to be engaged in livestock production (FAO et al., 2006). Moreover, there is a trend toward consolidation and intensification of the sector that occurs as livestock operations aim to increase production and efficiency (FAO et al., 2006; Randolph et al., 2007; Thornton, 2010).

Generally, it is difficult to predict or quantify the complex linkages between livestock production; economic, social, and cultural impacts; and human health and wellbeing (Perry & Grace, 2009). Overall, livestock production, including dairy farming, plays an important role in the economic stability and vitality at the individual, household, community, and national levels, in both lower‐ and higher‐income regions. There are also important social and cultural impacts associated with dairy production, although it is difficult to determine the net effect on human health and wellbeing.

## 9 Conclusions

This review attempts to provide a comprehensive overview of the linkages between the dairy sector and public health. The search strategies used at the outset captured a wide variety of studies from around the globe, including reviews; descriptive epidemiological studies, including ecological, cross‐sectional, case‐control, and cohort designs; and experimental research. Additional reports and articles were identified for inclusion through forward and backward citation searching and through supplemental searches. This review has attempted to present the breadth of evidence for both the positive and negative health effects associated with dairy production and consumption and aimed to include a representative sample of articles from the relevant literature. Previous reviews have examined a single facet of the issue (e.g., bovine zoonoses) but have not examined the broad spectrum of potential effects. To the authors' knowledge, this is the first review to examine the evidence for the full array of potential health impacts associated with dairy production and consumption (Figure 1).

Specifically, this review identified occupational hazards; environmental health effects; indirect ecosystem health effects; foodborne hazards; diet‐related harms and benefits; and indirect economic, social and cultural effects as important potential health impacts associated with dairy farming. Occupational and foodborne hazards, and diet‐related health harms and benefits have been covered more extensively in the existing literature than the other potential impacts. Indirect impacts that are difficult to quantify, especially ecosystem health impacts and social and cultural effects, are often overlooked. The public health burden depends on population exposure pathways, which operate at multiple spatial and temporal scales. Some of the potential public health impacts associated with dairy farming are concentrated in occupational groups and communities directly exposed to dairy cattle and rural environments. Other impacts affect consumers of dairy products through food safety and nutritional pathways, while certain effects occur at a regional or global scale through processes that may promote emerging infectious diseases and climate change.

### 9.1 Future Research

Generally, the potential indirect health effects associated with dairy production have not been as widely researched as direct health effects and ecosystem health, economic, social, and cultural impacts are often overlooked. The causal linkages between dairy production; environmental changes, economic, social, and cultural impacts; and human health and wellbeing are complex and difficult to quantify (Corvalan et al., 2005; Ingram, 2012; Perry & Grace, 2009). Additionally, the impacts of environmental change can be displaced in time and space and may be dependent upon a number of different modifying forces (Aron & Patz, 2001; Corvalan et al., 2005). However, the public health burden linked to indirect pathways may be substantial. For example, dairy production contributes to global climate change through the emission of GHGs, and climate change can severely impact human health, in terms of both direct health outcomes, including morbidity and mortality from extreme climate events (Corvalan et al., 2005; IPCC, 2014), and indirect outcomes through economic disruption; infrastructure damage; population displacement; loss of labor productivity; infectious disease risk alteration; changing availability of food, water, and materials; and other pathways (Corvalan et al., 2005; Foley et al., 2005; McMichael et al., 2006; Smith et al., 2014). Further research is required to improve our understanding of how dairy production affects public health through indirect pathways.

While occupational and foodborne hazards and diet‐related health harms and benefits have been more widely covered in the existing literature than other potential public health impacts, several knowledge gaps have been identified within those domains. Specifically, a number of epidemiological controversies were identified in the literature, such as the role of innate versus acquired immunity and questions about the etiologies of certain cancers and chronic disorders. A number of the identified knowledge gaps are highlighted below.

While zoonotic diseases have been extensively covered in the literature, additional research is required to clarify the role of dairy cattle in human zoonotic disease risk and to ascertain the precise transmission pathways by which humans are exposed to zoonotic pathogens. Additionally, the role of innate versus acquired immunity in the epidemiology of specific zoonoses is not well understood (Havelaar et al., 2009; Rothman & Mahon, 2004; Swift & Hunter, 2004) and requires additional research. Specifically, it has been hypothesized that long‐term exposure to certain pathogens may confer a degree of immunity (Havelaar et al., 2009). This would have important implications for occupational health, as many farm workers, veterinarians, and abattoir workers, and even rural communities, have frequent exposure to zoonotic pathogens (Havelaar et al., 2009; McDaniel et al., 2014; Toth et al., 2013).

AMR also represents a potential human health concern on dairy farms (Aitken et al., 2016; Call et al., 2008; Oliver et al., 2011; Tripathi & Tripathi, 2017). However, several knowledge gaps surrounding potential transmission routes between dairy cattle, humans, and the environment have been identified (Burgess & French, 2017). Specifically, more research is required on the prevalence of antimicrobial‐resistant pathogens carried by dairy cattle and the incidence of infection in humans who have had contact with dairy cattle (Burgess & French, 2017). Assessment of risk factors and transmission pathways would also improve understanding of AMR on dairy farms (Burgess & French, 2017).

Microbiological foodborne hazards associated with dairy consumption have also been well documented in the literature. However, questions remain about the public health burden of chemical and physical contaminants in milk and dairy products. Estimates for the number of illnesses caused by chemical agents are required for attribution studies. Additional research is also required to quantify the contribution of mycotoxins in milk and dairy products to the global burden of liver cancer. Furthermore, there are concerns that increased demand for animal feed and global climate change may lead to increases in mycotoxin occurrence in animal feed in some regions (Paterson & Lima, 2010; van Asselt et al., 2017). Therefore, projections of mycotoxin contamination of animal feed and subsequent human exposure through milk and dairy consumption under scenarios of climate change would help to predict future health risks and consequences.

With regard to diet‐related health harms and benefits associated with diary consumption, questions remain as to the role of dairy in the etiologies of certain cancers and chronic disorders. For example, naturally occurring hormones in milk may play a role in the etiology of certain forms of cancer (Malekinejad & Rezabakhsh, 2015; Pereira, 2014). Specifically, hormones in milk and dairy products may act as endocrine disrupters, and it has been hypothesized that exposure to steroid hormones through the consumption of milk and dairy products could promote the development of prostate, breast, and endometrial tumors (Malekinejad & Rezabakhsh, 2015). However, more research is needed to clarify the role of dairy consumption, and consequent exposure to steroid hormones, in the etiology of specific cancers (Malekinejad & Rezabakhsh, 2015). Additionally, dairy consumption has been associated with improved cognition (Crichton et al., 2010; Crichton et al., 2011; Hess et al., 2016; Park & Fulgoni, 2013; Yamada et al., 2003), but the mechanism by which dairy provides protection against dementia is unknown (Crichton et al., 2011; Kliem & Givens, 2011; Yamada et al., 2003), and more research is needed to determine how dairy consumption influences cognitive health.

There are a number of questions that remain unresolved, and future research should seek to expound upon the observed associations and clarify the potential linkages between dairy production and consumption and public health outcomes. Future research should also focus on regions heavily dependent on dairy farming, including both higher‐income areas with advanced, large‐scale production systems and lower‐income regions in which dairy farming is a critical poverty reduction tool.

### 9.2 Implications

As noted in section 1, the content of this review could be used to support improved decision making for the future development of the dairy sector from a public health perspective. There are a number of different types of decisions that this review could inform, ranging from strategic planning for optimal levels of dairy production and consumption at national and global levels to decisions about specific dairy conversion proposals compared with alternative land use options at national and regional levels. This review could also support decisions about the prioritization of potential health hazards associated with the dairy sector; resource allocation for the management of specific hazards associated with dairy production and consumption; and the identification of knowledge gaps that require further research to improve understanding and management of public health hazards. Ideally, the decision‐making process should include stakeholders who represent public health interests, and this review could support the broad consideration of such interests. Additionally, decision making is often made in the context of alternative options, such as different farming systems, alternative land uses, and different management choices, and this review provides information that can assist in identifying the consequences of the available options.

There are several available methods that can support decision‐making processes by providing systematic assessments of the public health impacts of dairy production and consumption at varying spatial and temporal scales. These methodologies include health impact assessment, health risk assessment, and environmental burden of disease analysis (Grout et al., 2018). Each of these processes entail a scoping phase to identify relevant hazards that could be supported by the contents of this review.

The impacts of dairy production will vary by the type of dairy farming system. The type of dairy farming system is, in turn, often related to a country's economic development. Smallholder dairy farming systems are typical in many lower‐income nations, while in higher‐income nations there has been a shift toward large, industrial dairy operations (FAO et al., 2006; Randolph et al., 2007; Thornton, 2010). Small‐scale dairy farming will have very different impacts from industrial scale dairy production.

A related consideration is the intensification of dairy production. In many parts of the world, dairy systems are undergoing rapid intensification, seeking to increase yields per unit area, typically through increased stocking rates and the use of off‐farm inputs (FAO et al., 2006). In areas where the current level of intensification is low, increases in dairy production may be easily accommodated by existing infrastructure. However, in areas where dairy farming has already intensified substantially, further attempts to increase production may contribute to a farming monoculture and undermine natural ecosystems (Aron & Patz, 2001; Corvalan et al., 2005; FAO et al., 2006), as evidenced by the ongoing debate over optimal levels of dairy production and alternatives to intensification occurring in several countries, including the United States and New Zealand (Baskaran et al., 2009; Clay et al., 2019).

Overall, this review of the current evidence can provide a starting point for stakeholders involved in the development of strategies and management policies for the dairy sector. Developing policies that achieve balance between potential public health harms and benefits and environmental and socioeconomic impacts is challenging and requires an understanding of the major health risks and exposure pathways associated with dairy production and consumption. As the global dairy sector increases production, exposure to a range of hazards must be weighed with the potential benefits to food security, nutrition, livelihoods, and economic opportunities in different settings. Lower‐ and higher‐income nations tend to have very different production systems, and the range of harms and benefits associated with dairy production can vary substantially by region. Therefore, international policy should attempt to reflect the potentially divergent needs of both lower‐ and higher‐income nations.

### 9.3 The Importance of an Integrated Approach

The main conclusion from this review is that decision makers need to take an integrated approach to considering complex policy questions, such as optimal development of the dairy sector. Agricultural production is inextricably linked to human health, and the environment and agricultural policy should not be approached with a silo mentality. Policies developed along conventional sectoral lines may not adequately address human health and environmental concerns. In order to address the potential public health impacts associated with dairy production and consumption, policy makers should consider cross‐sectoral approaches that reflect the complexity of agricultural systems and look for innovative solutions that leverage multiple benefits. For example, researchers have identified potential global health and environmental benefits associated with reducing the proportion of animal‐based foods in human diets (Springmann et al., 2016; Willett et al., 2019), especially in high‐income nations. Transitioning toward plant‐based diets that align with dietary guidelines could reduce global mortality by 6–10%, largely due to reductions in coronary heart disease, stroke, cancer, and type 2 diabetes related deaths (Springmann et al., 2016). Such a shift would also reduce GHG emissions by 29% to 70% and could provide economic benefits of U.S.$1–31 trillion (Springmann et al., 2016). Policies that can affect dietary shifts merit further consideration by decision makers, as evidence indicates that large‐scale dietary change could have significant health, environmental, and economic benefits (Springmann et al., 2016; Tilman & Clark, 2014; Westhoek et al., 2014; Willett et al., 2019).

## Conflict of Interest

The authors declare no conflicts of interest relevant to this study.

## Supporting information



Supporting Information S1
