# Supplementary material for: A Review of Potential Public Health Impacts Associated With the Global Dairy Sector
Source: Geohealth. 2020 Feb 13;4(2):e2019GH000213. doi: 10.1029/2019GH000213 (PMC7017588; doi:10.1029/2019GH000213)
Supplement: Supplementary file 1 — Supporting Information S1 [file GH2-4-e2019GH000213-s001.pdf]

AGU PUBLICATIONS

GeoHealth

Supporting Information for

A Review of Potential Public Health Impacts Associated with the Global Dairy Sector

Leah Grout[1], Michael G. Baker[1], Nigel French[2], and Simon Hales[1]

[1]Department of Public Health, University of Otago, Wellington 6021, New Zealand. [2]School of Veterinary Science, Hopkirk Research Institute, Massey University, Palmerston North 4474, New Zealand.

Contents of this file

Text S1 to S5

Introduction

Text S1 to S5 contain the search strategies used for the databases Medline, Embase, Scopus, Web of Science, PubMed, and Google Scholar, respectively. The search strategies were designed with the assistance of Michael Fauchelle at the University of Otago Wellington Medical and Health Sciences Library.

Text S1. Medline Search Strategy

Conducted 22 February 2017, retrieved 1,707 articles.

1. Dairying/
2. (dairying or (dairy adj (indust* or farm* or compan* or business* or production or herd* or cattle))).tw.
3. (milk* or “milk production”).tw.
4. 1 or 2 or 3
5. exp *Public Health/
6. (health* adj2 (population* or public* or impact* or effect* or outcome* or threat* or risk*)).tw.
7. 5 or 6
8. 4 and 7
9. Cattle/
10. (cow* or cattle or bovine*).tw.
11. 9 or 10
12. 8 and 11
13. limit 12 to English language
14. limit 13 to humans
15. limit 13 to animals
16. 13 not 15
17. 14 or 16
18. ae.xs
19. 1 and 18
20. limit 19 to English language
21. 17 or 20

Text S2. Embase Search Strategy

Conducted 22 February 2017, retrieved 579 articles.

1. Dairying/
2. (dairying or (dairy adj (indust* or farm* or company* or business* or production or herd* or cattle))).tw.
3. (milk* or “milk production”).tw.
4. 1 or 2 or 3
5. exp *Public Health/
6. (health* adj2 (population* or public* or impact* or effect* or outcome* or threat* or risk*))).tw.
7. 5 or 6
8. 4 and 7
9. Cattle/
10. (cow* or cattle or bovine*).tw.
11. 9 or 10
12. 8 and 11
13. limit 12 to english language
14. limit 13 to humans
15. limit 13 to animals
16. 13 not 15

17. 14 or 16
18. ae.xs.
19. 1 and 18
20. limit 19 to english language
21. 17 or 20

Text S3. Scopus Search Strategy

Conducted 27 March 2017, retrieved 265 articles.

((TITLE (dairying OR (dairy* W/1 (indust* OR farm* OR compan* OR business* OR production))) OR KEY (dairying OR (dairy* W/1 (indust* OR farm* OR compan* OR business* OR production))))) AND (TITLE-ABS-KEY (health* W/2 (population* OR public* OR impact* OR effect* OR outcome* OR threat* OR risk*))) AND (ALL (cow* OR cattle OR bovine*)) AND (EXCLUDE (SUBJAREA , "VETE")) AND (LIMIT-TO (LANGUAGE , "English"))

Text S4. Web of Science Search Strategy

Conducted 29 March 2017, retrieved 226 articles.

1. TITLE: ((dairying OR (dairy* NEAR/1 (indust* OR farm* OR company* OR business* OR production))))
   Indexes=SCI-EXPANDED, SSCI, A&HCI, CPCI-S, CPCI-SSH, ESCI, CCR-EXPANDED, IC Timespan=All years
2. TOPIC: ((dairying OR (dairy* NEAR/1 (indust* OR farm* OR company* OR business* OR production))))
   Indexes=SCI-EXPANDED, SSCI, A&HCI, CPCI-S, CPCI-SSH, ESCI, CCR-EXPANDED, IC Timespan=All years
3. TOPIC: (health* NEAR/2 (population* OR public* OR impact* OR effect* OR outcome* OR threat* OR risk*))
   Indexes=SCI-EXPANDED, SSCI, A&HCI, CPCI-S, CPCI-SSH, ESCI, CCR-EXPANDED, IC Timespan=All years
4. TOPIC: (cow* OR cattle OR bovine*)
   Indexes=SCI-EXPANDED, SSCI, A&HCI, CPCI-S, CPCI-SSH, ESCI, CCR-EXPANDED, IC Timespan=All years
5. #2 OR #1
   Indexes=SCI-EXPANDED, SSCI, A&HCI, CPCI-S, CPCI-SSH, ESCI, CCR-EXPANDED, IC Timespan=All years
6. #5 AND #3
   Indexes=SCI-EXPANDED, SSCI, A&HCI, CPCI-S, CPCI-SSH, ESCI, CCR-EXPANDED, IC Timespan=All years
7. #6 AND #4
   Indexes=SCI-EXPANDED, SSCI, A&HCI, CPCI-S, CPCI-SSH, ESCI, CCR-EXPANDED, IC Timespan=All years
8. #6 AND #4 **Refined by:** [excluding] **RESEARCH AREAS:** (VETERINARY SCIENCES)
   Indexes=SCI-EXPANDED, SSCI, A&HCI, CPCI-S, CPCI-SSH, ESCI, CCR-EXPANDED, IC Timespan=All years
9. #6 AND #4 **Refined by:** [excluding] **RESEARCH AREAS:** (VETERINARY SCIENCES) AND **LANGUAGES:** (ENGLISH)
   Indexes=SCI-EXPANDED, SSCI, A&HCI, CPCI-S, CPCI-SSH, ESCI, CCR-EXPANDED, IC Timespan=All years

Text S5. PubMed Search Strategy

Conducted 3 April 2017, retrieved 564 articles.

((((((dairying) OR ((dairy and (industr* or farm* or compan* or business* or production or herd*)))) AND Humans[Mesh] AND English[lang])) OR ((((milk* or "milk production"))) AND Humans[Mesh] AND English[lang])) AND ((("public health") OR ((health* adj2 (population* or public* or impact* or effect* or outcome* or threat* or risk*)))) AND Humans[Mesh] AND English[lang])) AND ((((cattle) OR cow*) OR bovine*) AND Humans[Mesh] AND English[lang]) Filters: Humans; English

Text S5. Google Scholar Search Strategy

Conducted 10 April 2017, retrieved 2,110 articles.

keyword: ((dairying OR (dairy AND (farm* OR production))) AND ("public health") AND (cattle OR cow* OR bovine*) -sheep -goat -camel -buffalo) since 2016, excluding patents and citations
